# Evolutionary Migration of the Disjunct Salt Cress *Eutrema salsugineum* (= *Thellungiella salsuginea*, Brassicaceae) between Asia and North America

**DOI:** 10.1371/journal.pone.0124010

**Published:** 2015-05-13

**Authors:** Xiao-Juan Wang, Da-Chuan Shi, Xin-Yu Wang, Juan Wang, Yong-Shuai Sun, Jian-Quan Liu

**Affiliations:** 1 MOE Key Laboratory for Bio-resources and Eco-environment, College of Life Science, Sichuan University, Chengdu, China; 2 Molecular Ecology Group, Key Laboratory of Arid and Grassland Ecology, School of Life Science, Lanzhou University, Lanzhou, China; Nanjing Forestry University, CHINA

## Abstract

*Eutrema salsugineum* (= *Thellungiella salsuginea* Brassicaceae), a species growing in highly saline habitats, is a good model for use in salt-stress research. However, its evolutionary migrations and genetic variations within and between disjunct regions from central Asia to northern China and North America remain largely unknown. We examined genetic variations and phylogeographic patterns of this species by sequencing ITS, 9 chloroplast (cp) DNA fragments (4379 bp) and 10 unlinked nuclear loci (6510 bp) of 24 populations across its distributional range. All markers suggested the high genetic poverty of this species and the limited number of genetic variations recovered was congruently partitioned between central Asia, northern China and North America. Further modelling of nuclear population-genetic data based on approximate bayesian computation (ABC) analyses indicated that the long-distance dispersals after the recent origin of *E*. *salsugineum* may have occurred from central Asia to the other two regions respectively within 20000 years. The fast demographic expansions should have occurred in northern China in a more recent past. Our study highlights the importance of using ABC analyses and nuclear population genetic data to trace evolutionary migrations of the disjunct distributions of the plants in the recent past.

## Introduction

Intercontinental disjunctions within the Northern Hemisphere have attracted much attentions of biogeography researchers [1–5]. Most disjunctions are at the generic level or among species groups and in only several cases, intercontinental populations in both Asia and North America were considered of the same species [1]. Previous researches were centered on phylogenetic constructions and fossil calibrations to trace diversification, migrations and vicariance at the generic level (e.g. [1]). In contrast, only fewer biogeographic works were designed to examine disjunctions of the same species with populations occurring in the different continents of the Northern Hemisphere. For example, the intercontinentally disjunct populations of *Phryma leptostachya* were estimated to diverge anciently within the early to middle Pliocene and this species migrated to eastern Asia via the Bering land bridge after its origin in North America [5]. The Bering bridge was suggested to be present for most of the Tertiary until the Pliocene and therefore provide migration routes for numerous groups or the same species with the current disjunctions in North America and eastern Asia [6] although the extreme cooling climates [2,7] probably cut off this migration route for many taxa during some glacial stages [3]. More recent migrations between these two continents after the closure of this bridge may have to rely on the long-distance dispersals by other mediators (for example, wind and bird) [4].

However, it is difficult to trace evolutionary migrations of such plants with recent disjunctions for two reasons. First, the commonly used DNA fragments (for example, ITS and chloroplast *rbc*L and *mat*K) whose genetic variations were used to construct phylogeny at the genus level, show no or fewer mutations within such species. Second, the previous approaches or methods are largely descriptive, without robust modeling and testing of alternative scenario. However, it should be noted that population genetic data based on sequencing multiple loci prove a powerful tool for overcoming these limitations and addressing related questions [8–9]. Sequence variation from multiple loci can generate more information to establish phylogeographic relationships between populations within a single species (e.g. [9]). In addition, coalescent analyses of population genetic data provide bases for identifying migration routes though modeling and testing of different hypothesized scenarios [10]. It is also likely that divergence times can be roughly estimated during coalescent analyses in the absence of a fossil record [9]. This estimation may make it possible to generate the temporal hierarchies for our understanding evolutionary migrations and demographic dynamics of disjunct plants [9,11].

*Eutrema salsugineum* (= *Thellungiella salsuginea* Brassicaceae) is disjunctly distributed from central Asia to North America. This species as well as its two close relatives, *E*. *halophilum* (= *Thellungiella halophila*) and *E*. *botschantzevii* (= *Thellungiella botschantzevii*) were together placed in *Thellungiella*, but now in *Eutrema* [12–13]. They are commonly called as salt cresses, known to tolerate high salt stress [14]. Like *Arabidopsis*, seeds of these species are very small and probably dispersed mainly by wind [12]. In addition, these species have favorable characteristics as abiotic stress model species [15]. In numerous laboratories across the world, they are becoming popular as an experimental model species for the elucidation of salt tolerance via molecular studies (e.g. [16–20]). These three species are together reported to occur in central Asia (Russia, Turkey, and western China) [13,21]. However, some ecotypes from northern China, representing those most commonly used in laboratories worldwide, have been described as *E*. *(Thellungiella) halophilum*. In fact, they should be ascribed to *E*. *salsugineum* while the true *E*. *halophilum* occurs only in central Asia [12]. These two species are closely related to each other although *E*. *halophilum* is outcrossing with pinnate leaves and fewer seeds while *E*. *salsugineum* is self-compatible with entire leaves and more seeds [13]. This re-circumscribed *E*. *salsugineum* occurs widely but disjunctly in saline habitats from central Asia to northern China and North America. The past collection records from the herbaria specimens suggest that the occurrence of this species in both central Asia and North America is infrequent and it is only limited to one and adjacent small localities. However, this species occurs commonly in northern China where it was collected from numerous localities. However, genetic diversity and phylogeographic history of this salt cress species remain largely unknown although similar studies on other model species, for example, *A*. *thaliana* as well as its close relatives, have received extensive attention and are clearly clarified (e.g. [22]).

In the present study, we aimed to trace evolutionary migration of *E*. *salsugineum* across central Asia, northern China and North America at the population level. We firstly examined the nuclear ITS variation between all samples and no variation at this DNA fragment confirmed that these samples originated from a common ancestor and should be taxonomically placed within one single species. We then sequenced nine maternally inherited chloroplast DNA fragments (totaling around 4000 bp in lenght), which are highly variable between intraspecific populations. We finally examined the sequence variation at 10 unlinked nuclear loci (around 6500 bp), all of which are known to be highly polymorphic within *Arabidopsis thaliana* (e.g. [23–24]). We used these sequence data to examine genetic diversity and construct the evolutionary migrations of this species. Unexpectedly, we recovered an extremely low genetic diversity in this salt cress. This genetic poverty suggests that long-distance dispersals mediated possibly by the wind may have leaded to disjunct distributions of this species from Asia to North America in the recent past. In addition, coalescent analyses of population genetic data from 10 nuclear loci identified the migration routes, divergence times and demographic histories of this salt cress across these disjunct regions.

## Materials and Methods

### Ethics Statement

All leave samples employed in this study were collected from *E*. *salsugineum* species and its two close relatives, *E*. *halophilum* and *E*. *botschantzevii* that are not endangered, and these plants grow in public area where no permission for collection of leaves is needed in China, Russia, Kazakhstan and Canada.

### Sampled populations

**Fig 1:**
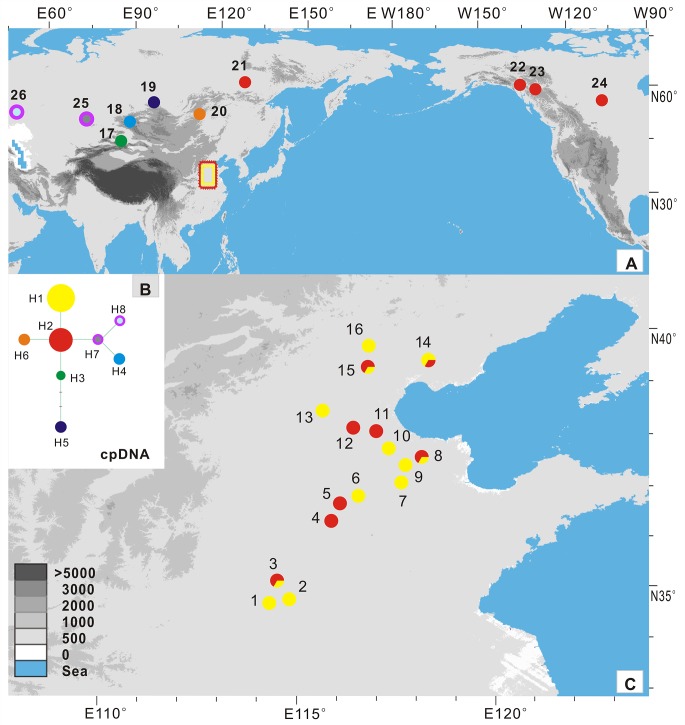
Sampling sites and cpDNA chlorotype frequencies. A. (The total region) and B. (Northern China). Sampling sites and cpDNA chlorotypes in each sampled population from *E*. *salsugineum* (1–24) and its relative species *E*. *halophila* (25) and *E*. *botschantzevii* (26). C. Network of the chlorotype. Circle size is proportional to chlorotype frequency. Pie charts indicate chlorotype frequency within each population.

We collected samples from 24 populations of *E*. *salsugineum*, one from Xinjiang, three from North America, four from Russia and the others mainly from northern China. Around 15 individuals for each population growing at least 50 m apart were collected in the field. However, only three to six individuals (a total of 99 individuals) for each population were used for the final phylogeographic analyses because our initial scanning of these individuals using most different markers failed to recover any variation between sampled individuals of each population. All leaves were dried and stored in silica gel. The latitude, longitude, and altitude of each population were recorded using a GPS, and these data were noted on a map using ArcMap in ArcGIS9.2 (Fig 1; S1 Table). We also sampled two close relatives, *E*. *halophilum* and *E*. *botschantzevii* (S1 Table).

### DNA extraction, PCR amplification and sequencing

We extracted DNA using the modified cetylcrimethyl ammonium bromide (CTAB) procedure described by Doyle and Doyle [25]. We amplified ITS primers following White et al. [26], 9 chloroplast (cp) DNA regions and 10 nuclear gene loci (primers see S2 and S3 Tables). These primers were designed according to the annotated genome [18] and the corresponding primers reported from *A*. *thaliana* (e.g. [23–24]). These unlinked nuclear loci are evenly distributed in the different scaffolds of this salt cress and display high variations between populations of *A*. *thaliana*. All PCRs were performed in a 25 μL volume, including 10–40 ng total DNA, 50 mm Tris-HCl, 1.5 mm MgCl_2_, 250 μg/mL BSA, 0.5 mm dNTPs, 2 μm primer, and 0.75 U of *Taq* polymerase. We used the following thermal protocol: initially 6 min at 94°C, followed by 37 cycles of 40 s at 94°C, 40 s of annealing at 48°C to 60°C, 1 min at 72°C and a final 7 min extension at 72°C. All PCR products were further purified using a TIANquick Midi Purification Kit according to the recommended protocol (TIANGEN). Sequencing reactions were conducted with the same PCR primers using an ABI Prism Bigdye Terminator version 3.1 Cycle Sequencing Kit. We conducted the following sequencing by an ABI 3730XL DNA Analyzer. All the sequences have been submitted to GenBank (Accessions no: KP208685-KP208704, KP219004-KP219019, KP453985-KP453987). All obtained sequences were aligned using CLUSTAL X version 1.81 [27] and double-checked manually.

### Analyses of sequence variation and population structure

We used DnaSP v. 5.00 [28] to estimate basic population genetic parameters for the cpDNA and nuclear loci examined: *S*, the number of segregating sites; *N*_h_, the number of haplotypes; *H*_e_, the haplotype diversity; and the nucleotide diversity (π and θ) [29–31]. Haplotype networks were constructed using the Median-Joining model implemented in the program NETWORK 4.0 [32]. Tajima’s D statistic [33], Fu and Li’s D* and F* [34], as well as Fay and Wu’s H [35] statistic for the site frequency spectrum, were calculated for all nuclear loci combined. We tested departure from the standard neutral model by comparing the observed values of the summary statistics with their expected distributions based on 10,000 coalescent simulations.

We used STRUCTURE version 2.3.2 [36] to assess the correspondence between geographical grouping and genotypic clustering. To infer the structure of the sampled populations in STRUCTURE, the likelihood of each number of clusters, *K*, where 1≤*K*≤10, was assessed and allowance made for the correlation of allele frequencies between clusters. Twenty runs were performed with a burn-in of 100,000 and then 1,000,000 iterations. The most likely number of clusters was estimated using the original method described by Pritchard et al. [37] and the Δ*K* statistics given in Evanno et al. [38]. Second, to estimate the variance component and to partition the variation within and between populations, we used analysis of molecular variance (AMOVA) implemented in ARLEQUIN 3.0 [39].

### Tests for expansion based on nDNA sequences

To test for population expansion, based on nDNA sequences, mismatch distributions of the observed number of nucleotide differences between pairs of nDNA sequences were computed using Arlequin version 3.0 [39]. We used a total of 1000 parametric bootstrap replicates based on segregating sites to generate an expected distribution according to a model of sudden demographic expansion [40]. We also used the sum of squared deviation (SSD) as a statistic to test the validity of the expansion model, with *P* values calculated as the proportion of simulations that produced a larger SSD than the observed SSD. We calculated the raggedness index (RAG) and its significance to quantify the smoothness of the observed mismatch distribution. Estimation and testing were conducted using Arlequin version 3.0 [39] with 1,000 bootstrap replicates for Fu’s F_S_. As suggested, this statistic is very sensitive to recent demographic expansion for which large, negative values are typically obtained [41]. To assess further the demographic history of the species, we also used LAMARC v2.2 [42], a coalescent-based method that takes account of genealogical relationships among haplotypes, to estimate the exponential population growth rate parameter ‘g’. All MCMC runs produced similar results, so here we present the results for the longest runs, which were composed of three replicates of 10 initial chains and two long final chains. The initial chains were performed using 10000 samples and a sample interval of 50 (500, 000 steps), with a burn-in of 50, 000 (100, 000 steps).

### Tests of alternative scenarios for evolutionary migration and divergence by DIYABC

**Fig 2:**
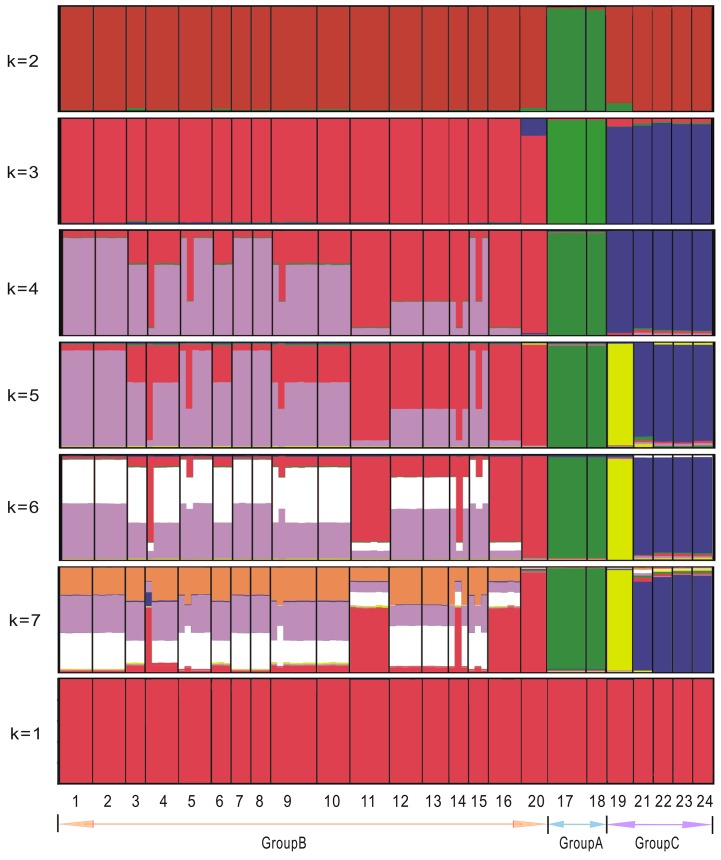
STRUCTURE analyses of the sampled populations. 24 sampled *E*. *salsuginea* populations and individuals based on nuclear loci. Bar plots showing the proportion of inferred co-ancestry from Bayesian population assignment tests. Results are shown for K = 1 to K = 7. Population numbers in the Group A, B and C referred to those in the Fig 1. These three groups were further used for ABC analyses.

ABC is as a powerful approach to select obtain the most suitable demographic history by statistically testing alternative hypotheses [10]. We used the software DIYABC v1.0.4.39 [43–44] to select the evolutionary scenario of *E*. *salsugineum* based on population genetic data from 10 loci. The assorted population genetic data were simulated under four hypothesized scenarios with population divergence, population size change and admixture [43]. Three groups (Group A, B and C) were defined based on the results from the STRUCTURE analyses (Fig 2). Moreover, initial LAMARC and mismatch distribution analyses indicated that populations from northern China experienced a common rapid population expansion. Therefore, we added population size change models to these four scenarios. For the ABC analyses, parameter values were set from the minimum to maximum range of priors. We used the number of haplotypes, number of segregating sites and mean pairwise difference as one-sample summary statistics. We chose pairwise differences (W) and (B) as two-sample summary statistics to compare between the observed and simulated datasets. We conducted one million simulations for each scenario, and selected the most likely scenario through the posterior probabilities with both direct approach and logistic regression methods. In addition, we also evaluated the most scenario by a principal component analysis (PCA) using the option “model checking” in DIYABC. We assumed a generation time of one year for *E*. *salsugineum* as observed for all populations.

During the ABC analyses, we used the method of Ikeda et al. [45] to estimate the mutation rate (μ) across the sampled loci. We calculated the average mutation rate according to the formula: μ = μCHS × K_Total_/K_S_ × L, where L is the length of the locus, K_Total_/K_S_ is the ratio of the number of all substitutions per substitution site (K_Total_) to the number of synonymous substitutions per synonymous site (K_S_) and μCHS is the substitution rate per synonymous site per year of the CHS gene in Brassicaceae, estimated to 1.5 × 10^–8^ substitutions per site per year [46]. The final used mean rate was 6.55 × 10^–6^ (S4 Table), substitutions per year per locus (= 9.3 × 10^–9^, substitutions per year per site). We also used the mutation rate (μ = 5–10.0 × 10^–9^ per site per year) recorded for other genera of the same family [47] for ITS sequence to estimate the divergence of *E*. *salsugineum*-*E*. *halophilum* and *E*. *botschantzevii* [48].

## Results

### ITS sequence variation

All ITS sequences showed no variation with the sampled 99 individuals of *E*. *salsugineum* (S1 Table). In addition, no variation was found between *E*. *salsugineum* and *E*. *halophilum*. However, they together differed from the third species *E*. *botschantzevii* with three mutations (S5 Table). According the ITS mutation rate (μ = 5–10.0 × 10^**–9**^ per site per year) [47] was adopted, *E*. *salsugineum*—*E*. *halophilum* together diverged from *E*. *botschantzevii* between 240 and 480 thousand years ago.

### cpDNA sequence variation

We sequenced 9 cpDNA fragments (S2 Table) and a total of 4379 bp from 99 individuals in 24 populations (Fig 1; S1 Table). The sequenced nucleotides amounted to one million. Only one of the nine cpDNA fragments, *psb*A-*trn*H, was found to be polymorphic (S6 Table). We also sequenced the *psb*A-*trn*H fragment from two populations in species *E*. *halophilum* and *E*. *botschantzevii* respectively (S1 and S6 Tables). At this locus, five substitutions and one indel and two reverse complements with 7 bp differentiated all individuals into eight haplotypes (Fig 1B; S6 Table). Two haplotypes (H1 and H2) with two mutations were widespread in northern China. In addition, H2 was also fixed in three populations in Northern America and one northeastern Russian population. Three haplotypes (H3-H5) with two mutations, one indel and two reverse complements were fixed in three populations (17–19) from central Asia respectively. H6 from the central Russian population was close to the highly frequent H2. It is worth noting, the two other haplotypes (H7-H8) from two populations in species *E*. *halophilum* and *E*. *botschantzevii* included one indel shared with the haplotype (H4) from central Asia Altai population and one mutation fixed in *E*. *botschantzevii*. The nucleotide diversities between all *E*. *salsugineum* individuals at the *psb*A-*trn*H locus and all cpDNA loci were estimated to be θ = 0.00345 and π = 0.00281, and θ = 0.00032 and π = 0.00026, respectively (Table 1). We failed to detect any significant departure from neutral evolution for any of the nuclear loci or cpDNA (S7 Table).

**Table 1:** Nucleotide variation, haplotype diversity for 10 nuclear loci and 9 cpDNA from *E*. *salsuginea* populations.

Locus	N	L	S	θ_wt_	π_t_	θ_wa_	π_a_	θ_sil_	π_sil_	N_h_	H_d_	Rm
**nDNA**												
*COP*	99	517	2	0.00085	0.00075	0	0	0.00129	0.00146	3	0.411	0
^¶^ *DET*	99	545	0	0	0	0	0	0	0	1	0	0
^¶^ *FAH*	99	462	0	0	0	0	0	0	0	1	0	0
*CHS*	99	931	1	0.00021	0.00041	0.00037	0.00072	0	0	2	0.381	0
^¶^ *F3H*	99	496	0	0	0	0	0	0	0	1	0	0
*PGIC*	99	800	2	0.00048	0.00068	0	0	0.00060	0.00084	3	0.516	0
*RPS1*	99	953	5	0.00102	0.00084	0.00052	0.00066	0.00291	0.00155	5	0.602	0
*RPS3*	99	468	1	0.00041	0.00072	0	0	0.00079	0.00137	2	0.338	0
^¶^ *HKT*	99	711	0	0	0	0	0	0	0	1	0	0
^¶^ *ThSOS1*	99	627	0	0	0	0	0	0	0	1	0	0
Average	99	651	1.1	0.00030	0.00034	0.00009	0.00014	0.00056	0.00052	2	0.225	0
Aligned	99	6510	11	0.00036	0.00042	-	-	-	-	9	0.856	1
^§^ **cpDNA**												
*psbA-trnH*	99	405	7	0.00345	0.00281	0	0	0.00393	0.00321	6	0.632	1
Average	99	486.6	0.78	0.00038	0.00031	0	0	0.00044	0.00036	-	0.070	-
Aligned	99	4379	7	0.00032	0.00026	0	0	-	-	6	0.632	1

N, sample size; L, length in base pairs; S, number of segregating sizes; π, nucleotide diversity (π_t_, π_a_ and π_sil_ are in total locus, nonsynonymous site and silent site respectively); θ, Watterson’s parameter (θ_wt_, θ_wa_ and θ_sil_ are in total locus, nonsynonymous site and silent site respectively); N_h_, number of haplotypes; He, Nei’ s haplotype diversity; Rm, minimum number of recombinant events

¶, no polymorphisms

-, no data

**§**, 8 cpDNA fragments with no variations (see S3 Table) were not shown.

### Nuclear DNA sequence variation across 10 loci

We further sequenced 10 unlinked nuclear loci (S3 Table) and the total length of these nuclear fragments was around 6510 bp. We found that five loci were polymorphic. One synonymous mutation was recovered at one locus (*CHS*) and a total of ten non-coding mutations were recovered at four loci (*RPS1*, *COP*, *PGIC* and *RPS3*). Pairwise sequence diversity (θ) ranged from 0 to 0.00102 while π ranged from 0 to 0.00084 (Table 1). The average nucleotide diversity across all 10 loci was estimated to be θ = 0.00036 and π = 0.00042. Haplotype genealogies were further constructed for each locus by NETWORK (S1 Fig). The posterior probability of *K*, *L*(*K*) and Δ*K* were computed by means of STUCTURE analysis, using the runs with highest probability for each value of *K*. Bayesian clustering estimated the uppermost hierarchical level of structure at *K* = 10 groups, in the absence of a priori classification. However, the largest break in *L*(*K*) was located at *K* = 2 and *K* = 3 based on the modal value of Δ*K* as an indicator of the uppermost level of hierarchical structure [38] (S2 Fig). When *K* = 2, two populations from central Asia (populations 17 and 18) (Group A) were separated from other populations (Fig 2). When *K* = 3, two more groups were identified: Groups B comprised one central Russia population (20, Buriatia) and 16 populations (1–16) from northern China while Group C consisted of one population (19) from central Asia and other four populations from Russia and North America (21–24).

### Genetic differentiation between regions and regional expansion tests

Analyses of both cpDNA haplotype distributions suggested distinct regional differentiation between central Asia, northern China and North America. AMOVA analyses also supported this inferences (results not shown). We further placed three populations from Russia into Group B (20, 1–16) and Group C (19, 21–24) as inferred from STUCTURE analyses and examined their genetic differences with the Group A from central Asia (two populations 17–18). We found that 76.82% of the nuclear variations were partitioned between these three groups, and 20.22% between populations (S8 Table).

**Fig 3:**
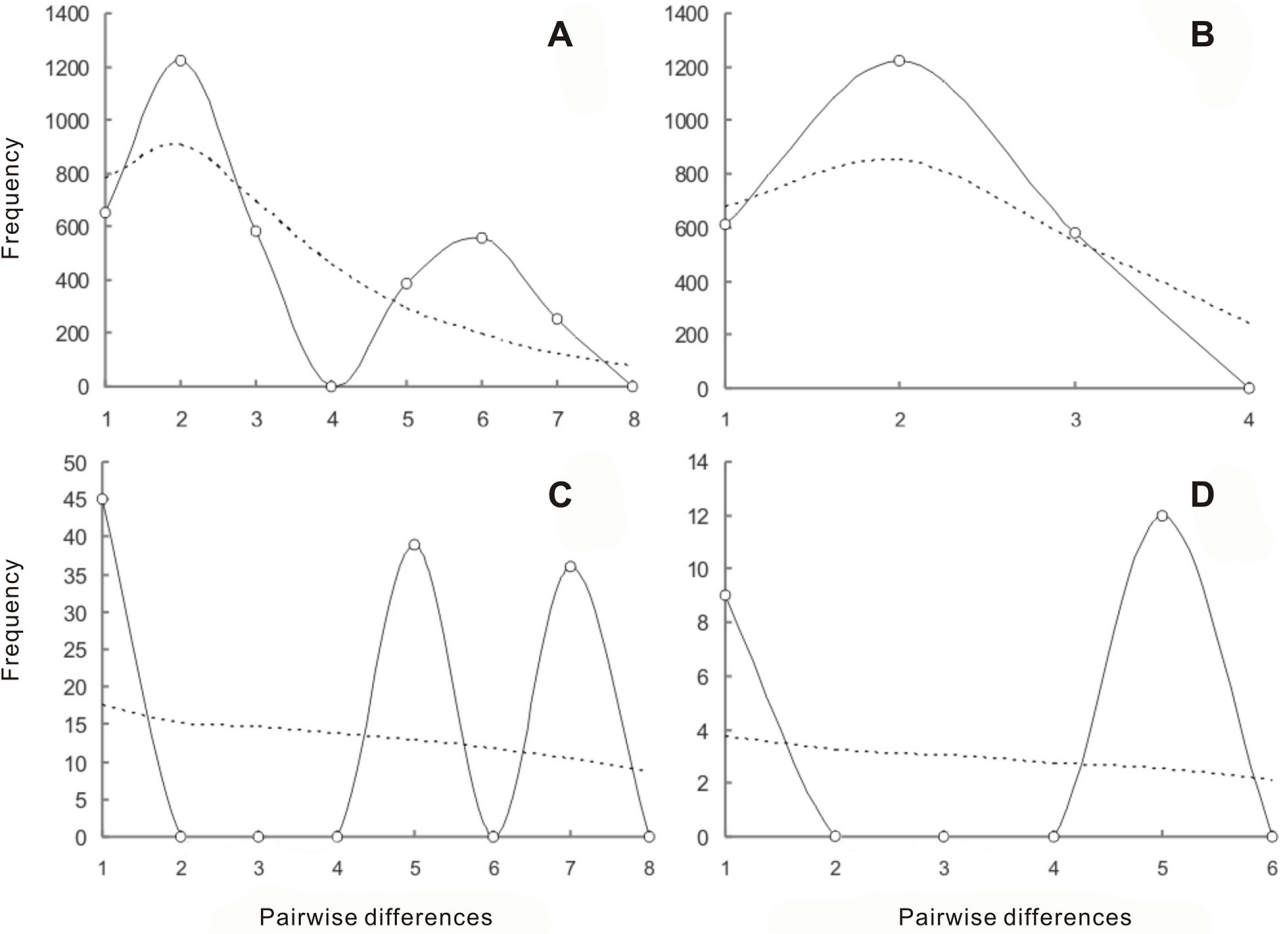
Results of the mismatch distribution analyses for four groups. A. All examined populations. B. Populations from northern China and Buriatia. C. Seven populations excluding those occurring in northern China and Buriatia. D. Five populations from North America and Russia (equaling to Group C). Dotted lines refer to the distributions expected for an expanding population, while the continuous lines represent the observed distributions of pairwise differences among samples.

The mismatch analyses based on nuclear data for 17 populations for Group B from northern China plus Buriatia suggested a distinct population expansion (Fig 3B). Further analyses of the variance (SSD) and raggedness index (RAD) suggested that the curves did not differ significantly from those of distributions expected based on a model of sudden population expansions (Table 2). In addition, the growth rate parameter ‘g’, derived from LAMARC tests [49], obviously supported the population expansion of this group (g = 351.89) (Table 2). However, we failed to detect expansion for the other two groups of populations possibly due to the fewer recovered mutations.

**Table 2:** Demographic tests based on mismatch distribution and LAMARC.

Group	*θ*_0_	*θ*_1_	SSD (*P* value)	RAG (*P* value)	Growth rate (g)
24 populations	0	4.659	0.026 (0.30)	0.06 (0.385)	-547.7
17 populations^a^	0	99999	0.0141 (0.02)	0.1336 (0.002)	351.89
7 populations^b^	0	5.59	0.1135 (0.054)	0.2978 (0.02)	-809
2 populations^c^	-	-	-	-	-
5 populations^d^	0.002	2.139	0.0768 (0.228)	0.2131 (0.299)	-1838.185

*θ*_0_ and *θ*_1_ are pre-expansion and post-expansion populations sizes; SSD, sum of squared deviations; RAG, the Harpending’s Raggedness index

a, with populations from northern China and Buriatia (Group B)

b, without populations from northern China and Buriatia

c, with populations from Xinjiang and Altai, both of which have the same sequence (Group A)

d, with populations from Tuva,Yakutsk and Canada (Group C)

-, no data.

### Favored scenarios for evolutionary migration based on ABC simulations

**Fig 4:**
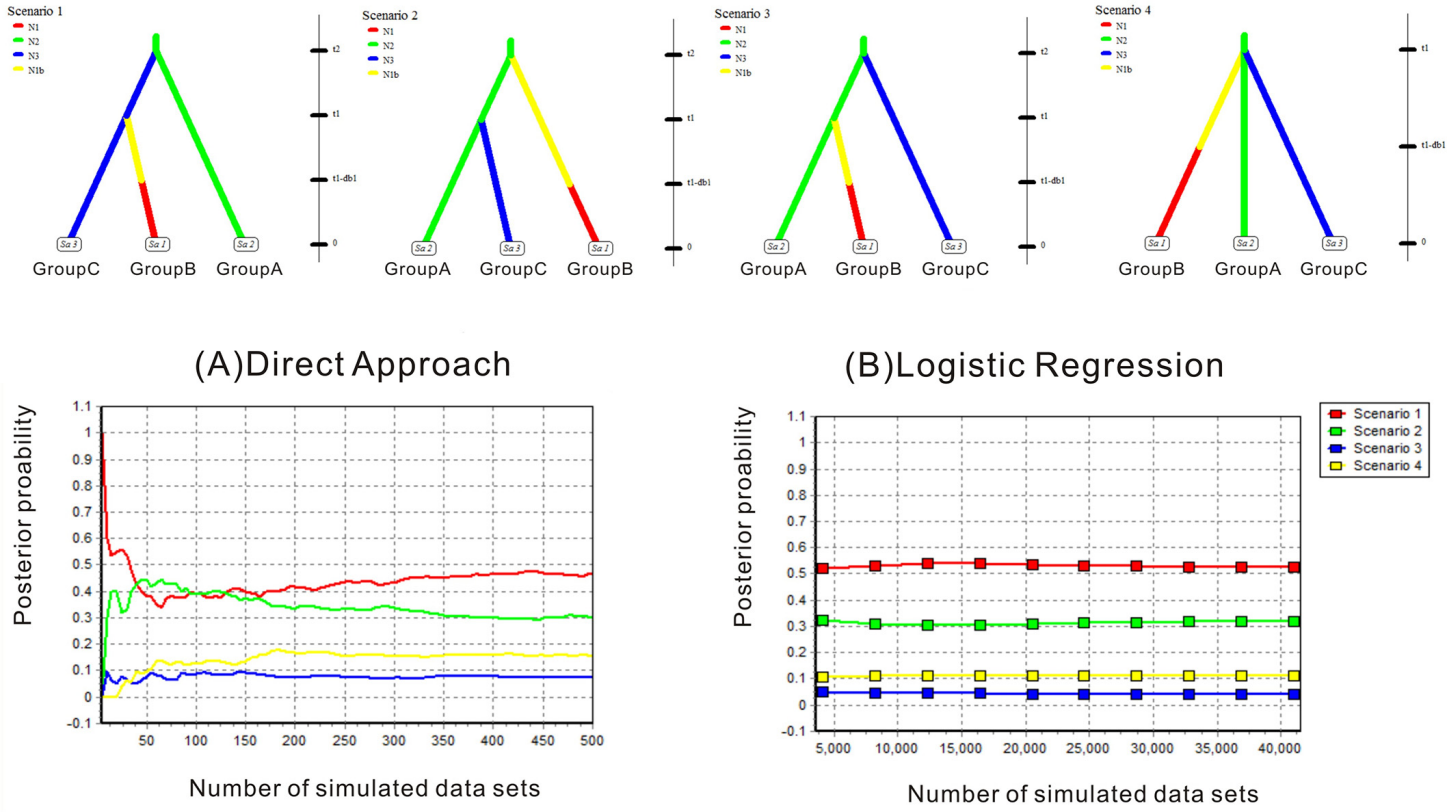
Estimation of the relative likelihood of four scenarios for the origin of *E*. *salsuginea*. The DIYABC graphs showed the four best supported scenarios tested together. For each scenario, different colors indicated corresponding population sizes (Ne). Graphs indicate the relative likelihoods of the four scenarios above compared by (A) direct approach, and (B) logistic regression on the 1% (41,000) and 400 closest simulated data sets, respectively. The graphs illustrated that Scenario 1 is the best.

**Fig 5:**
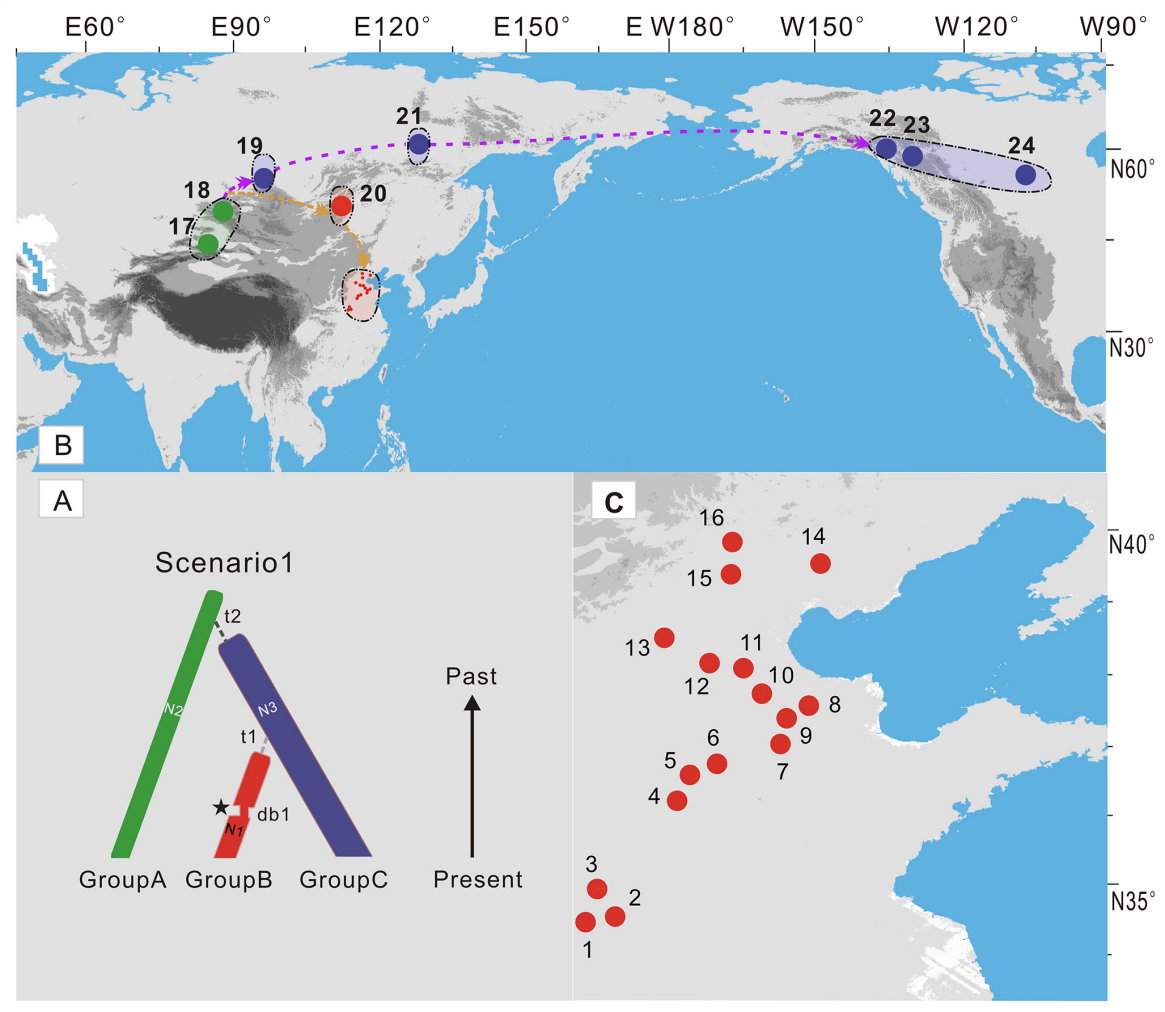
The migration history of *E*. *salsugineum*. A. The mostly favored scenario (scenario 1). Groups A, B and C equal to those defined by STUCTURE (*K* = 3) analyses. Width of population bars is proportional to effective size (Ne) and the fluted patterns (pentagon) in Group B indicates the expansion. B. Migration routes of *E*. *salsugineum* from central Asia to northern China and North America. Pie charts shows population assignment to groups based on structure analyses (*K* = 3). C. Populations came from northern China.

To better understand evolutionary migration, we used ABC to simulate 4 most probable scenarios (Fig 4). The highest posterior probability was favored for scenario 1 (direct estimate 0.4680 [95% CI: 0.0360–0.9054]; logistic regression 0.5250 [95% CI: 0.5068–0.5431]) (Fig 4; S9 Table). When the mutation rate 6.55 × 10^–6^ substitutions per year per locus (S4 Table) was used to scale the demographic parameters from DIYABC, the first divergence between central Asia and others was estimated to have occurred 23 thousand years ago (kya) while Group B (mainly in northern China) diverged from Group C mainly distributed North America around 11 kya (Fig 5B; S10 Table). In addition, after this divergence, Group B expanded greatly in northern China within the Holocene.

## Discussion

In the present study, we used population genetic data from nine cpDNA fragments and ten nuclear loci to examine genetic variations within and between populations of *E*. *salsugineum* and further to outline its evolutionary migrations across disjunct regions from central Asia to North America. Our studies suggested the extremely low genetic diversity of this species by both datasets and these consistent genetic patterns indicated that this species may have experienced rapid migration to reach its current ranges since its origin in the recent past. We tested the different scenarios of the migration routes and inferred the most likely migration routes and timescales.

### Low genetic diversity

We analyzed patterns of genetic variation using a sequencing survey of 9 cpDNA segments, 10 nuclear loci from 24 populations of *E*. *salsugineum*. The nucleotide diversities across all cpDNA fragments were estimated to be θ = 0.00032 and π = 0.00026 and across all nuclear loci to be θ = 0.00036 and π = 0.00042. In contrast to the initial expectation, we found extremely low genetic diversity in this species. The average nucleotide diversity (π) of *A*. *thaliana* from 11 cpDNA segments was estimated to be 0.00169. Its nucleotide diversity (θ) observed from 334 nuclear loci has been estimated to be 0.00896 [50] and from 11 loci to be 0.0241 [51]. Other studies based on fewer loci or on a single locus have also revealed several-fold higher genetic diversity in this mesic species than the salt cress we studied here. For example, the nucleotide diversities (π) at the *RPS1* and *PGIC* loci have been estimated to be 0.0126 and 0.00380 for the mesic *A*. *thaliana* [23–24] and only 0.00084 and 0.00068 for *E*. *salsugineum*. In addition, we found no genetic variation in the other five loci (Table 1). However, nucleotide diversity (π) at these loci, for example, *F3H* and *FAH*, was high in *A*. *thaliana*, with values of 0.00700 and 0.00300 [52]. In fact, to our knowledge, *E*. *salsugineum* is the most depauperate herb species in which nucleotide diversity has been studied by means of multiple loci (S11 Table), although in a few extremely endangered species with narrow distributions lower or no genetic diversity has been found using other molecular markers (e.g. [53]). The poor genetic diversity of *E*. *salsugineum* based on cpDNA (H = 0.070) (Table 1) is similar to that of one regionally symbolic circum-Mediterranean pine, in which, only four haplotypes were found after scanning 12 cpDNA loci in 34 populations across the species’ distribution range (H = 0.019) [54].

The greatly lower genetic diversity in *E*. *salsugineum* compared with the mesic *A*. *thaliana* contrasts to species examined in comparative studies in Israel’s “Evolution Canyon” [55]. In fact, modeling tests have also suggested that under extreme stress, genetic diversity will be greatly reduced, rather than increased, as under the stimuli of arid conditions [56]. High environmental pressure may have triggered strong stabilizing and purifying selection affecting *E*. *salsugineum*. In order to adapt to highly saline habitats, this species may have developed specific traits, with genetic diversity decreasing as all populations stabilized on these traits [56]. Any mutation (allele) is likely to be deleterious to the survival of such species in the arid habitats, so would disappear through purifying selection [57]. In addition, strong purifying selection on a locus, i.e. the purging of deleterious variants, will result in the occasional removal of linked variation, producing a decrease in the level of variation surrounding the locus [58]. In other words, background selection under such a scenario may purge non-deleterious alleles close to deleterious alleles, further decreasing the genetic diversity of the species [59]. However, the reduced genetic diversity acted by natural selections should be centered on a few loci, rather than at the genomic level [56]. The congruent genetic patterns with low diversity illustrated by all markers suggested that such a lack of genetic diversity across its wide but disjunct distributions may have mainly derived from the rapid migration in the recent past [60]. In addition, the total genetic diversity at cpDNA fragments and nuclear DNA loci was found to be mainly partitioned between the three geographical groups and genetic differentiation between or within these regions, in fact, are still very low (S8 Table).

### Recent origin, long-distance migration and rapid expansion in northern China

Since all sampled individuals of *E*. *salsugineum* and *E*. *halophilium* have the same ITS sequence, which differs from another species *E*. *botschantzevii* with only three mutations. Based on the mutation rate recorded for other genera of the same family [47], *E*. *salsugineum* and *E*. *halophilium* together diverged from *E*. *botschantzevii* 240–480 thousand years ago (kya). The further divergence between *E*. *salsugineum* and *E*. *halophilium* should have occurred at a later stage. These divergences fall within the middle Pleistocene when central Asia became drier than before and desertification and salinization began to develop and expand [61]. This habitat change may have triggered origin of *E*. *salsugineum* and its divergence with the closely related species in central Asia [12]. After its origin, it migrated out of central Asia at around 23 Kya and later colonized northern China and North America by two migratory routes at 11 Kya as inferred from ABC modeling of the nuclear population genetic data. However, only did it reach northern China, it expanded and reached widespread distributions there (Fig 5B,C; S10 Table). This expansion was well confirmed by the mismatch distribution, LAMARC and ABC analysis (Figs 3 and 4; Table 2). The widespread distribution of two cpDNA haplotypes in most populations (Fig 1) also suggested recent colonization and rapid expansion in this region.

Our analyses indicate that this species migrate from central Asia to northern China and North America very recently. It remains unknown what might have served as this important mediator for long-distance dispersals. Because seeds of this species, like those of *A*. *thaliana*, are very small, it is highly likely that the frequent sandstorms since the late Pleistocene [62] might have carried its seeds to northern China and North America through Russian regions. These recent dispersals and subsequent expansions may partly account for the extremely low diversity within this widely distributed species. The intercontinental disjunction of *E*. *salsugineum* differs distinctly from the previous studies (e.g. [1–4,63]), because it occurred probably very recently. This suggested that intercontinental disjunctions of plants within the Northern Hemisphere were more complex than previously expected and the recent long-distance dispersals mediated possibly by wind might also lead to such an intercontinental distribution[4]. More studies of such recent dispersals are needed to fully understand the diverse mechanisms for the intercontinental disjunctions in the Northern Hemisphere.

## Conclusions

Our results obviously suggested that *E*. *salsugineum* originated and diverged from its two closely related species very recently. It started its long-distance dispersal to northern China and North America very recently. When this species arrived at the northern China, it may have expanded rapidly in the recent past. The recent origin and long-distance fast colonization together resulted in the low genetic diversity of this species despite the fact that this species seem to be distributed in a large scale from central Asia to North America. Such a genetic homogeneity across the total species’ range corroborate the idea that this species will prove a good model for salt-stress research (e.g. [16–17]) because its genetic pool is highly homologous.

## Supporting Information

S1 Fig Haplotype genealogies for five nuclear loci. (TIF)

S2 Fig Bayesian inference for the best number of clusters (K). (TIF)

S1 Table Origins and soil conditions of the *E*. *salsuginea* populations and two close relatives populations. (DOC)

S2 Table Primers for amplifying and sequencing the 9 cpDNA fragments. (DOC)

S3 Table Primers and gene functions of the 10 sequenced nuclear loci. (DOC)

S4 Table The mutation rate μ for each nuclear gene was estimated from K_Total_/K_S_. (DOC)

S5 Table Variable sites of the ITS fragment and the three genotypes from three closely related species. (DOC)

S6 Table Variable sites of the only polymorphic cpDNA fragment *psb*A-*trn*H. (DOC)

S7 Table Neutrality tests of the 10 nuclear loci and cpDNA. (DOC)

S8 Table AMOVA analyses for all genetic variations based on cpDNA and nuclear DNA sequences. (DOC)

S9 Table Description of the four scenarios used in the approximate Bayesian. (DOC)

S10 Table Demographic parameters obtained by DIYABC 1.0.4.39. (DOC)

S11 Table The total nucleotide diversity (π and θ) of a range of plant species. (DOC)

S12 Table GenBank accession numbers of all sequences included in this study. (DOC)
