# Supplementary figures and images for: Evolutionary Migration of the Disjunct Salt Cress Eutrema salsugineum (= Thellungiella salsuginea, Brassicaceae) between Asia and North America
Source: PLoS One. 2015 May 13;10(5):e0124010. doi: 10.1371/journal.pone.0124010 (PMC4430283; doi:10.1371/journal.pone.0124010)

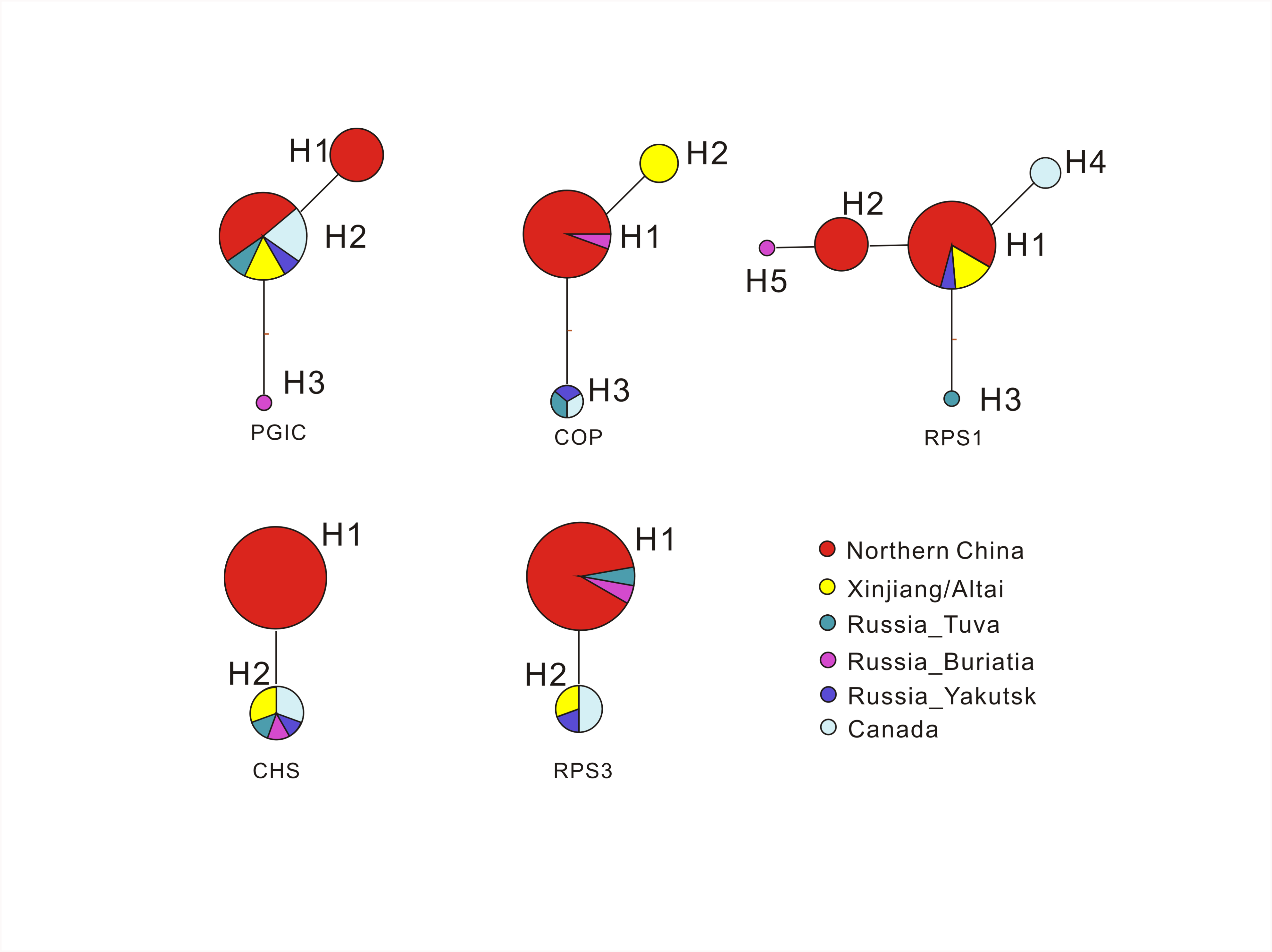

Supplement: S1 Fig — (TIF) [file pone.0124010.s001.tif]

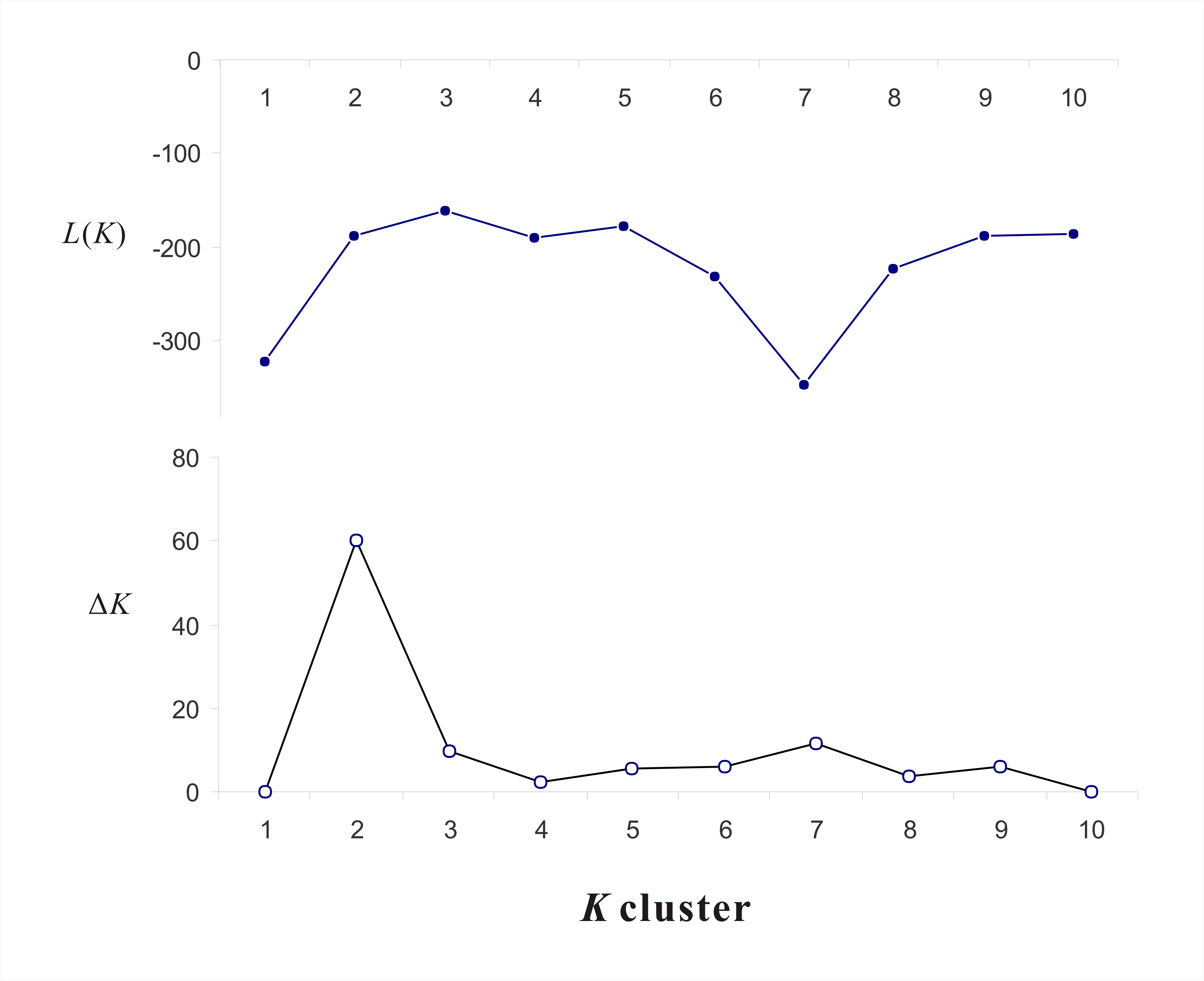

Supplement: S2 Fig — (TIF) [file pone.0124010.s002.tif]
