# Supplementary material for: Evolutionary Migration of the Disjunct Salt Cress Eutrema salsugineum (= Thellungiella salsuginea, Brassicaceae) between Asia and North America
Source: PLoS One. 2015 May 13;10(5):e0124010. doi: 10.1371/journal.pone.0124010 (PMC4430283; doi:10.1371/journal.pone.0124010)
Supplement: S1 Table — (DOC) [file pone.0124010.s003.doc]

**S1 Table. Origins and soil conditions of the *E. salsuginea* populations and two close relatives populations.**

| **N.** | **Population code** | **Latitude** (N) | **Longitude** (E) | **Altitude**(m) |
| --- | --- | --- | --- | --- |
| 1 | Kaifeng, HN | 34°47′53′′ | 114°18′6′′ | 75 |
| 2 | Lankao, HN | 34°49′23′′ | 114°48′56′′ | 67 |
| 3 | Fengqiu, HN | 34°02′30′′ | 114°24′46′′ | 73 |
| 4 | Liaocheng, SD | 36°27′23′′ | 115°58′46′′ | 37 |
| 5 | Shiping, SD | 36°36′ | 116°20′ | 29 |
| 6 | Qihe, SD | 36°46′59′′ | 116°45′25′′ | 23 |
| 7 | Boxing, SD | 37°8′57′′ | 118°07′32′′ | 7 |
| 8 | Dongying, SD | 37°26′2′′ | 118°40′8′′ | 2 |
| 9 | Binzhou, SD | 37°22′52′′ | 117°57′53′′ | 9 |
| 10 | Huimin, SD | 37°29′20′′ | 117°30′15′′ | 11 |
| 11 | Yanshan, HB | 38°03′ | 117°13′28′′ | 12 |
| 12 | Botou, HB | 38°4′58′′ | 116°34′22′′ | 15 |
| 13 | Raoyang, HB | 38°14′6′′ | 115°44′11′′ | 22 |
| 14 | Leting, HB | 39°25′28′′ | 118°54′24′′ | 12 |
| 15 | Qingguang, TJ | 39°11′45′′ | 117°3′12′′ | 11 |
| 16 | Wuqing, TJ | 39°22′58′′ | 117°2′17′′ | 8 |
| 17 | Manasi, XJ | 44°18′10′′ | 86°12′41′′ | 467 |
| 18 | Altai, Russian | 49°59′ | 88°40′ | 354 |
| 19 | Tuva, Russian | 51°-55° | 95°37′ | 1982 |
| 20 | Buriatia, Russian | 51°-55° | 112°24′ | 1164 |
| 21 | Yakutsk, Russian | 62° | 129°43′ | 127 |
| 22 | Yukon, Canada | 60°51′ | 135°43′W | 969 |
| 23 | Cracker Creek, Canada | 59°42′ | 133°24′W | 1680 |
| 24 | Dillibrough, Canada | 56°07′ | 106°20′W | 469 |
| 25 | *E. halophilum* | 50°47′ | 75°42′ | - |
| 26 | *E. botschantzevii* | 51° | 45°-46° | - |

N., Number of the population; N.1-24 is the sampled *E. salsuginea* populations, N.25-26 is two close relatives populations: *E. halophilum, E. botschantzevii* respectively; HB, Hebei; HN, Henan; SD, Shandong; TJ, Tianjin; XJ, Xingjiang; Vouchers have been deposited in the herbarium of Molecular Ecology Group, Key Laboratory for Bio-resources and Eco-environment, Sichuan University (Chengdu, China). -, no data.
