# Supplementary material for: Evolutionary Migration of the Disjunct Salt Cress Eutrema salsugineum (= Thellungiella salsuginea, Brassicaceae) between Asia and North America
Source: PLoS One. 2015 May 13;10(5):e0124010. doi: 10.1371/journal.pone.0124010 (PMC4430283; doi:10.1371/journal.pone.0124010)
Supplement: S2 Table — (DOC) [file pone.0124010.s004.doc]

**S2 Table. Primers for amplifying and sequencing the 9 cpDNA fragments.**

| **Gene** | **PCR primers** | **Annealing temperature (**°C**)** | **Variation** |
| --- | --- | --- | --- |
| *psbA-trnH* | F: CGAAGCTCCATCTACAAATGG  R: ACTGCCTTGATCCACTTGGC | 50 | 7 |
| *trnD-trnT* | F: ACCAATTGAACTACAATCCC  R: CTACCACTGAGTTAAAAGGG | 48 | 0 |
| *ndhF1* | F: TGACAAAACATGTGCCGATT  R: AAAAAGCATCTATGTAACCACGA | 57 | 0 |
| *ndhF2* | F: TTACTTTTGAAGGACATTTAAACACTT  R: TTCCATCAATGATTCGTTTATCA | 58 | 0 |
| *trnL-trnF* | F: GTCCGTTTGACACCTTACCC  R: TATCCTGGCCATTACCGAAG | 59 | 0 |
| *trnV* | F: GTAGAGCACCTCGTTTACAC  R:CTCGAACCGTAGACCTTCTC | 55 | 0 |
| *rpoC1* | F: GGGTCTCACTTTCAAATTGCAT  R: CAATTTGGGATGTCCTCGAT | 59 | 0 |
| *trnL* | F: GGATTGAGCCTTGGTATGGA  R: CCTCACGATTTTAAAAGTCAACG | 60 | 0 |
| *rbcL* | F: ATGTCACCAAAAACAGAGACTAAAG  R: TGGATTACAAGTAATCAATCGTATC | 50 | 0 |
