# Supplementary material for: Evolutionary Migration of the Disjunct Salt Cress Eutrema salsugineum (= Thellungiella salsuginea, Brassicaceae) between Asia and North America
Source: PLoS One. 2015 May 13;10(5):e0124010. doi: 10.1371/journal.pone.0124010 (PMC4430283; doi:10.1371/journal.pone.0124010)
Supplement: S3 Table — (DOC) [file pone.0124010.s005.doc]

**S3 Table. Primers and gene functions of the 10 sequenced nuclear loci**.

| **locus** | **Function of the gene** | **PCR Primers** | **Annealing temperature (**°C**)** | **Variation** |
| --- | --- | --- | --- | --- |
| *COP* | Regulator related to phytochome and cryptochrome. | 5’-ACGAGGCAGGAAGCAAGTGT-3’  5’-CACTGTGAGACCCACAAAGTTCTT-3’ | 54 | 2 |
| *DET* | Regulator related to phytochome and cryptochrome. | 5’-GGTTCAGTTTTTGGATCGACA-3’  5’-GGAGGGACTTTGTGACTGACA-3’ | 55 | 0 |
| *FAH* | Encoding an enzyme of the phenylpropanoid pathway. | 5’-AACCGGCCTGCAACTATAGC-3’  5’-ATGAGCCACGGCTAAGTCAA-3’ | 58 | 0 |
| *CHS* | Glucosinolate biosynthesis and possible insect resistance.  Important in secondary metabolism. | 5’-CTTCATCTGCCCGTCCATCAAACC-3’  5’-GCGTTCTGTTTAGAGAGGAACGC-3’ | 55 | 1 |
| *F3H* | Important in secondary metabolism. Encoding an enzyme of the phenylpropanoid pathway. | 5’-ACAAGCTCCGTTTCGACATG-3’  5’-GTGGTCGCCGAGATTGAC-3’ | 58 | 0 |
| *PGIC* | Encoding an enzyme in glucose metabolism. | 5’-TCGAACCCGGGAGAGGTAGACCA-3’  5’-TGCTGTCAGCACTAATCTTGCG-3’ | 55 | 2 |
| *HKT* | Encoding a sodium transporter expressed in xylem parenchyma cells | 5'- CTCCCCCTTTTCTTCCTTTACTT-3'  5'- ACACAAAGACGAACGGAGAGTAA-3' | 53 | 0 |
| *RPS1* | Resistance to pseudomonas syringae AvrRpt2 | 5’-ACTCGTAATCAAAACCGAACTAAAT-3’  5’-CTGAGCAAACTAACTATTAGTTAAT-3’ | 51 | 5 |
| *RPS3* |  | 5’-AAATATACTCGTTTCTTGGACCAAG-3’  5’-TGGCGATGCATAAACCTTGCTATAA-3’ | 57 | 1 |
| *ThSOS1* | CAP transcription factors, related to salt tolerance | 5’-GTGCAGGGAACGAAATCAAC-3’  5’-TCAGGACATACGCTCCAAGT-3’ | 55 | 0 |
| Total |  |  |  | 11 |
