# Supplementary material for: Evolutionary Migration of the Disjunct Salt Cress Eutrema salsugineum (= Thellungiella salsuginea, Brassicaceae) between Asia and North America
Source: PLoS One. 2015 May 13;10(5):e0124010. doi: 10.1371/journal.pone.0124010 (PMC4430283; doi:10.1371/journal.pone.0124010)
Supplement: S4 Table — (DOC) [file pone.0124010.s006.doc]

**S4 Table. The mutation rate μ for each nuclear gene was estimated from K_Total_/K_S._**

| **Locus** | **L** | **K_Total_** | **K_S_** | ***μ*(10**^-^**^6^)** |
| --- | --- | --- | --- | --- |
| *CHS* | 931 | 0.00042 | 0 | - |
| *COP* | 517 | 0.001171 | 0.001457 | 6.23 |
| *PGIC* | 800 | 0.000707 | 0.000822 | 10.32 |
| *RPS1* | 953 | 0.000866 | 0.001554 | 7.96 |
| *RPS3* | 468 | 0.000709 | 0.001384 | 3.6 |
| Average | 733.8 | 0.000775 | 0.001045 | 7.02 |
| Geomean | 703.0299 | 0.000734 | 0.001267 | 6.55 |

**L**, the length of the locus; **K_Total_,** the number of all substitions per substitution site; **K_S_,** the number of synonymous substitions per synonymous site.
