# Supplementary material for: Evolutionary Migration of the Disjunct Salt Cress Eutrema salsugineum (= Thellungiella salsuginea, Brassicaceae) between Asia and North America
Source: PLoS One. 2015 May 13;10(5):e0124010. doi: 10.1371/journal.pone.0124010 (PMC4430283; doi:10.1371/journal.pone.0124010)
Supplement: S5 Table — (DOC) [file pone.0124010.s007.doc]

**S5 Table. Variable sites of the ITS fragment and the three genotypes from three closely related species**.

| **Species** | **ITS** | | |
| --- | --- | --- | --- |
|  | 399 | 425 | 481 |
| *Eutrema (Thellungiella) salsuginea* | - | G | G |
| *Eutrema (Thellungiella) halophila* | - | G | G |
| *Eutrema botschantzevii* | C | A | A |
