# Supplementary material for: Evolutionary Migration of the Disjunct Salt Cress Eutrema salsugineum (= Thellungiella salsuginea, Brassicaceae) between Asia and North America
Source: PLoS One. 2015 May 13;10(5):e0124010. doi: 10.1371/journal.pone.0124010 (PMC4430283; doi:10.1371/journal.pone.0124010)
Supplement: S6 Table — (DOC) [file pone.0124010.s008.doc]

**S6 Table. Variable sites of the only polymorphic cpDNA fragment *psb*A*-trn*H.**

| **Species** | **Haplotype** | ***psb*A*-trn*H** | | | | | | | |
| --- | --- | --- | --- | --- | --- | --- | --- | --- | --- |
|  |  | 43 | 108 | 129 | 156 | 162 | 208 | 277 | 304 |
| *E. salsuginea* | H1 | A | C | TGAATTT | A | TTTCTAT | A | - | C |
|  | H2 | A | C | TGAATTT | A | TTTCTAT | A | - | A |
|  | H3 | A | C | TGAATTT | A | ATAGAAA | A | - | A |
|  | H4 | A | C | TGAATTT | A | TTTCTAT | C | A | A |
|  | H5 | A | C | AAATTCA | G | ATAGAAA | A | A | A |
|  | H6 | A | T | TGAATTT | A | TTTCTAT | A | - | A |
| *E. halophila* | H7 | A | C | TGAATTT | A | TTTCTAT | A | A | A |
| *E.* *botschantzevii* | H8 | G | C | TGAATTT | A | TTTCTAT | A | A | A |

It showed the eight haplotypes identified from all sampled individuals*.* Sequences are numbered from the 5′-end to the 3′-end for each region.
