# Supplementary material for: Evolutionary Migration of the Disjunct Salt Cress Eutrema salsugineum (= Thellungiella salsuginea, Brassicaceae) between Asia and North America
Source: PLoS One. 2015 May 13;10(5):e0124010. doi: 10.1371/journal.pone.0124010 (PMC4430283; doi:10.1371/journal.pone.0124010)
Supplement: S7 Table — (DOC) [file pone.0124010.s009.doc]

**S7 Table. Neutrality tests of the 10 nuclear loci and cpDNA.**

| **Locus** | **D** | **D*** | **F*** | **H** |
| --- | --- | --- | --- | --- |
| **nDNA** |  |  |  |  |
| *COP* | 0.21364 | 0.48986 | 0.56643 | 0.22099 |
| *DET* | - | - | - | - |
| *FAH* | - | - | - | - |
| *CHS* | 1.11515 | 0.48986 | 0.78908 | -0.7474 |
| *F3H* | - | - | - | - |
| *PGIC* | 0.46002 | 0.68452 | 0.78268 | 0.27541 |
| *HKT* | - | - | - | - |
| *RPS1* | -0.37153 | 1.04776 | 0.68710 | 0.5848 |
| *RPS3* | 0.8557 | 0.48986 | 0.69866 | 0.24675 |
| *ThSOS1* | - | - | - | - |
| Aligned | 0.34951 | 1.50776 | 1.34224 | 0.90806 |
| **cpDNA** |  |  |  |  |
| *psb*A-*trn*H | -0.43070 | 1.21097 | 0.77458 | -0.99175 |
| Aligned | -0.43070 | 1.21097 | 0.77458 | -0.99175 |

D, Tajima’s D statistic; H, Fay and Wu’s H; D*****and F*****, Fu and Li’s D***** and Fu and Li’s F*****.
