# Supplementary material for: Evolutionary Migration of the Disjunct Salt Cress Eutrema salsugineum (= Thellungiella salsuginea, Brassicaceae) between Asia and North America
Source: PLoS One. 2015 May 13;10(5):e0124010. doi: 10.1371/journal.pone.0124010 (PMC4430283; doi:10.1371/journal.pone.0124010)
Supplement: S8 Table — (DOC) [file pone.0124010.s010.doc]

**S8 Table. AMOVA analyses for all genetic variations based on cpDNA and nuclear DNA sequences.**

| **Source of variation** | **SS** | **VC** | **Variation (%)** | **Fixation index** |
| --- | --- | --- | --- | --- |
| **nDNA genotypes** |  |  |  |  |
| **(i) All populations** |  |  |  |  |
| Among populations | 128.1 | 1.34011 | 95.58027 |  |
| Within populations | 4.648 | 0.06197 | 4.41973 | F_ST_=0.95580 |
| **(ii) Two groups** |  |  |  |  |
| Among groups | 30.725 | 1.54981 | 57.75114 | F_CT_=0.57751 |
| Among populations within groups | 97.375 | 1.07182 | 39.93971 | F_SC_=0.94534 |
| Within populations | 4.648 | 0.06197 | 2.30915 | F_ST_=0.97691 |
| **(iii) Three groups** |  |  |  |  |
| Among groups | 54.734 | 1.83295 | 76.82558 | F_CT_=0.76826 |
| Among populations within groups | 36.281 | 0.48249 | 20.22291 | F_SC_=0.87264 |
| Within populations | 4.648 | 0.07042 | 2.95150 | F_ST_=0.97048 |
| **cpDNA haplotypes** |  |  |  |  |
| **(i) All populations** |  |  |  |  |
| Among populations | 51.535 | 0.53632 | 93.78266 |  |
| Within populations | 2.667 | 0.03556 | 6.21734 | F_ST_=0.93783 |
| **(ii) Two groups** |  |  |  |  |
| Among groups | 4.842 | 0.28867 | 34.94200 | F_CT_=0.34942 |
| Among populations within groups | 46.694 | 0.50197 | 60.75415 | F_SC_=0.93385 |
| Within populations | 2.667 | 0.03556 | 4.30385 | F_ST_=0.95696 |
| **(iii) Three groups** |  |  |  |  |
| Among groups | 13.990 | 0.26580 | 36.75857 | F_CT_=0.36759 |
| Among populations within groups | 37.546 | 0.42174 | 58.32424 | F_SC_=0.92225 |
| Within populations | 2.667 | 0.03556 | 4.91719 | F_ST_=0.95083 |

SS, Sum of squares; VC, Variance components (Two groups: Xinjiang and Altai (GroupA) vs. the others (GroupB+C); Three groups: Xinjiang and Altai (GroupA) vs. northern China and Buriatia (GroupB) vs. Tuva,Yakutsk and Canada (GroupC)).
