# Supplementary material for: Evolutionary Migration of the Disjunct Salt Cress Eutrema salsugineum (= Thellungiella salsuginea, Brassicaceae) between Asia and North America
Source: PLoS One. 2015 May 13;10(5):e0124010. doi: 10.1371/journal.pone.0124010 (PMC4430283; doi:10.1371/journal.pone.0124010)
Supplement: S9 Table — (DOC) [file pone.0124010.s011.doc]

**S9 Table. Description of the four scenarios used in the approximate Bayesian.**

|  | **Direct approach** | | **Logistic regression** | |
| --- | --- | --- | --- | --- |
| **Scenario** | **Posterior probability** | **95% CI (lower-upper)** | **Posterior probability** | **95% CI (lower-upper)** |
| 1 | 0.4680 | (0.0360,0.9054) | 0.5250 | (0.5068,0.5431) |
| 2 | 0.3020 | (0.0000,0.7044) | 0.3199 | (0.3026,0.3372) |
| 3 | 0.0740 | (0.0000,0.3035) | 0.0418 | (0.0369,0.0467) |
| 4 | 0.1560 | (0.0000,0.4741) | 0.1133 | (0.1036,0.1231) |

Computation analysis in DIYABC 1.0.4.39 to test the origin and the differentiation time among species. Each row contained four scenarios from Figure. 4 (scenario 1-4), The relative posterior probabilities and 95% confidence intervals for each scenario were computed via the logistic regression on 1% of the closest data sets to the observed data.
