# Supplementary material for: Evolutionary Migration of the Disjunct Salt Cress Eutrema salsugineum (= Thellungiella salsuginea, Brassicaceae) between Asia and North America
Source: PLoS One. 2015 May 13;10(5):e0124010. doi: 10.1371/journal.pone.0124010 (PMC4430283; doi:10.1371/journal.pone.0124010)
Supplement: S10 Table — (DOC) [file pone.0124010.s012.doc]

**S10 Table. Demographic parameters obtained by DIYABC 1.0.4.39**.

| **Parameter** | **mean** | **median** | **mode** | **quantile 2.5%** | **quantile 5%** | **quantile 95%** | **quantile 97.5%** |
| --- | --- | --- | --- | --- | --- | --- | --- |
| N_1_ | 3070 | 3070 | 3180 | 218 | 403 | 5700 | 5850 |
| N_2_ | 496 | 491 | 82 | 33 | 6 | 948 | 975 |
| N_3_ | 6160 | 6160 | 6490 | 1170 | 1710 | 11400 | 11700 |
| db1(generation) | 7810 | 7910 | 12800 | 476 | 881 | 14300 | 14700 |
| N1b | 1850 | 1780 | 141 | 96 | 177 | 3750 | 3880 |
| t1(generation) | 11100 | 11300 | 9250 | 2460 | 3400 | 18000 | 18600 |
| t2(generation) | 23200 | 24000 | 27800 | 12300 | 14100 | 29500 | 29700 |
| mutation rate  (per site per generation) | 9.3E-09 (about 6.55 × 10^-6^ substitutions per year per locus) | | | | | | |

the parameter of the geometric distribution to generate multiple stepwise mutations.
