# Supplementary material for: Evolutionary Migration of the Disjunct Salt Cress Eutrema salsugineum (= Thellungiella salsuginea, Brassicaceae) between Asia and North America
Source: PLoS One. 2015 May 13;10(5):e0124010. doi: 10.1371/journal.pone.0124010 (PMC4430283; doi:10.1371/journal.pone.0124010)
Supplement: S11 Table — (DOC) [file pone.0124010.s013.doc]

**S11 Table. The total nucleotide diversity (π and θ) of a range of plant species.**

| **Species** | **No. of loci** | **Length**  (bp) | **Species-wide** | **Nuclear diversity** | **References** |
| --- | --- | --- | --- | --- | --- |
| *Thellungiella salsuginea* | 9 chloroplast loci | 4,379 | Yes | π=0.00026  θ=0.00032 | The present work |
|  | 10 nuclear loci | 6,510 | Yes | π=0.00042  θ=0.00036 | The present work |
| *Arabidopsis thaliana* | 11 chloroplast loci | 8750 | Yes | π= 0.00169 | [1] |
|  | 11 nuclear loci | 7,959 | Yes | θ=0.0241±0.004  π=0.0219±0.005 | [2] |
|  | 334 nuclear loci | 13,9038 | 22 | θ=0.00896 | [3] |
| *A. halleri* | 24 nuclear loci | 12,678 | 12 | θ=0.00900 | [4] |
|  | 8 nuclear loci | 7,821 | Yes | π=0.00810 | [5] |
| *A. lyrata* ssp*. petraea* | 8 nuclear loci | 7,821 | Yes | π=0.01160 | [5] |
| *A.lyrata*ssp*.lyrata* | 3 nuclear loci | 3,300 | Yes | π=0.00170 | [5] |
| *Helianthus annuus* (wild) | 9 nuclear loci | 8,207 | 16 | π=0.01280  θ=0.0144 | [6] |
| *H. annuus*(Primitive) | 9 nuclear loci | 8,207 | 16 | π=0.00560  θ= 0.0072 | [6] |
| *Hordeum vulgare* ssp. *spontaneum* | 18 nuclear loci | 25,030 (aligned) | Yes | π=0.00750 | [7] |
| *H. vulgare* (wild) | 4 nuclear loci | 7,156 | Yes | π= 0.00235-0.00336 | [8] |
| *H. petiolaris* | 18 nuclear loci | 11,927 | 10 | π_s_=0.03318  θ_s_=0.04394 | [9] |
| *Solanum peruvianum* | 8 nuclear loci | 10,214 | Yes | π=0.01290  θ=0.01810 | [10] |
| *S. chilense* | 8 nuclear loci | 10,214 | Yes | π=0.01100  θ=0.01270 | [10] |
| *Sorghum bicolor* | 95 nuclear loci | 29,186 | Yes | π=0.00225 | [11] |
| *Oryza rufipogon* | 5 nuclear loci | 4,087 | Yes | π_s_=0.0080  θ_s_=0.0131 | [12] |
|  | 2 chlorolplast loci | 1,320 | Yes | π_s_=0.00215  θ_s_=0.0016 | [12] |
| *O. nivara* | 5 nuclear loci | - | Yes | π_s_=0.0063  θ_s_=0.0101 | [12] |
|  | 2 chlorolplast loci | - | Yes | π_s_= 0.0025  θ_s_=0.00145 | [12] |
| *O. sativa* L. ssp. *indica* | Genome-wide | - | Yes | π=0.00670 | [13] |
| *Zea mays* L. ssp*. mays* | 18 nuclear loci | 6,935 | Yes | π= 0.00630 | [14] |
| *Z. mays* L. ssp*. parviglumis* | 21 nuclear loci | 14,420 | Yes | θ=0.0096±32 | [15] |
| *Cardamine nipponica* | 10 nuclear loci | 7,068 | 19 | π= 0.0013 | [16] |
| *Aquilegia formosa* | 9 nuclear loci | 7,337 | 12 | π=0.004-0.006 | [17] |
| *A. pubescens* | 9 nuclear loci | 7,337 | 4 | π=0.004-0.006 | [17] |
| *Populus balsamifera* | 3 nuclear loci | 1,827 | 8 | π=0.0030  θ=0.0019 | [18] |
| *P. tremula* | 77 nuclear loci | 42,350 | 2 | π= 0.0042  θ=0.0048 | [19] |
| *Juniperus przewalskii* | 8 nuclear loci | 5,084 | Yes | π= 0.00193  θ=0.00175 | [20] |
| *Picea likiangensis* | 16 nuclear loci | 10,827 | Yes | π_s_=0.00930 | [20] |
| *Pinus densata* | 7 nuclear loci | 3,040 | Yes | π= 0.0086  θ=0.0101 | [21] |
| *P. tabuliformis* | 7 nuclear loci | 3,040 | Yes | π= 0.0085  θ=0.0107 | [21] |
| *P. yunnanensis* | 7 nuclear loci | 3,040 | Yes | π= 0.0067  θ=0.0055 | [21] |

-, no data.

**References**

1. Yin P, Kang JQ, He F, Qu LJ, Gu HY (2010) The origin of populations of *Arabidopsis thaliana* in China, based on the chloroplast DNA sequences. BMC Plant Biol 10: 22.
2. Shepard KA, Purugganan MD (2003) Molecular population genetics of the Arabidopsis CLAVATA2 region, the genomic scale of variation and selection in a selﬁng species. Genetics 163: 1083-1095.
3. Schmid KJ, Ramos-Onsins S, Ringys-Beckstein H, Weisshaar B, Mitchell-Olds T (2005) A multilocus sequence survey in *Arabidopsis thaliana* reveals a genome-wide departure from a neutral model of DNA sequence polymorphism. Genetics 169: 1601-1615.
4. Heidel AJ, Ramos-onsins SE, Wang WK, Chiang ZY, Mitchell-olds T (2010) Population history in *Arabidopsis halleri* using multilocus analysis. Mol Ecol 19: 3364-3379.
5. Ramos-Onsins SE, Stranger BE, Mitchell-Olds T, Aguade M (2004) Multilocus analysis of variation and speciation in the closely related species *Arabidopsis halleri* and *A. lyrata*. Genetics 166: 373-388.
6. Liu A, Burke JM (2006) Patterns of nucleotide diversity in wild and cultivated sunflower. Genetics 173: 321-330.
7. Morrell PL, Lundy KE, Clegg MT (2003) Distinct geographic patterns of genetic diversity are maintained in wild barley (*Hordeum vulgare* ssp. *spontaneum*) despite migration. Proc Natl Acad Sci U S A 100: 10812-10817.
8. Caldwell KS, Russell J, Langridge P, Powell W (2006) Extreme population-dependent linkage disequilibrium detected in an inbreeding plant species, *Hordeum vulgare*. Genetics 172: 557-567.
9. Strasburg JL, Rieseberg LH (2008) Molecular demographic history of the annual sunflowers *Helianthus annuus* and *H. Petiolaris*-large effective population sizes and rates of Long-term gene flow. Evolution 62: 1936-1950.
10. Arunyawat U, Stephan W, [Städler T](http://www.ncbi.nlm.nih.gov/pubmed?term=Städler T%5BAuthor%5D&cauthor=true&cauthor_uid=17675653) (2007) Using Multilocus Sequence Data to Assess Population Structure, Natural Selection, and Linkage Disequilibrium in Wild Tomatoes. Mol Biol Evol 24: 2310-2322.
11. Hamblin MT, Mitchell SE, White GM, Gallego J, Kukatla R, Wing RA et al. (2004) Comparative population genetics of the panicoid grasses, sequence polymorphism, linkage disequilibrium and selection in a diverse sample of *Sorghum bicolor*. Genetics 167: 471-483.
12. Zheng XM, Ge S (2010) Ecological divergence in the presence of gene flow in two closely related Oryza species (*Oryza rufipogon* and *O. nivara*). Mol Ecol 19: 2439-2454.
13. [Yu J](http://www.ncbi.nlm.nih.gov/pubmed?term=Yu J%5BAuthor%5D&cauthor=true&cauthor_uid=11935017)1, [Hu S](http://www.ncbi.nlm.nih.gov/pubmed?term=Hu S%5BAuthor%5D&cauthor=true&cauthor_uid=11935017), [Wang J](http://www.ncbi.nlm.nih.gov/pubmed?term=Wang J%5BAuthor%5D&cauthor=true&cauthor_uid=11935017), [Wong GK](http://www.ncbi.nlm.nih.gov/pubmed?term=Wong GK%5BAuthor%5D&cauthor=true&cauthor_uid=11935017), [Li S](http://www.ncbi.nlm.nih.gov/pubmed?term=Li S%5BAuthor%5D&cauthor=true&cauthor_uid=11935017), Liu B et al. (2002) A draft sequence of the rice genome (*Oryza sativa* L. ssp. *indica*). Science 296: 79-92.
14. Ching A, Caldwell KS, Jung M, Dolan M, Smith OS, Tingey S et al. (2002) SNP frequency, haplotype structure and linkage disequilibrium in elite maize inbred lines. BMC Genet 3: 19.
15. Tenaillon MI, Sawkins MC, Long AD, Gaut RL, Doebley JF, Gaut BS (2001) Patterns of DNA sequence polymorphism along chromosome 1 of maize (*Zea mays* ssp. *mays* L). Proc Natl Acad Sci U S A 98: 9161-9166.
16. Ikeda H, Fujii N, Setoguchi H (2009) Application of the isolation with migration model demonstrates the Pleistocene origin of geographic differentiation in *Cardamine nipponica* (Brassicaceae), an endemic Japanese alpine plant. Mol Biol Evol 26: 2207-2216.
17. Cooper EA, Whittall JB, Hodges SA, Nordborg M (2010) Genetic variation at nuclear loci fails to distinguish two morphologically distinct species of *Aquilegia.* PLoS ONE 5: e8655.
18. Breen AL, Glenn E, Yeager A, Olson MS (2009) Nucleotide diversity among natural populations of a north American poplar (*Populus balsamifera*, Salicaceae). New Phytol 182: 763-773.
19. Ingvarsson PK (2008) Multilocus patterns of nucleotide polymorphism and the demographic history of *Populus tremula*. Genetics 180: 329-340.
20. [Li Y](http://www.ncbi.nlm.nih.gov/pubmed?term=Li Y%5BAuthor%5D&cauthor=true&cauthor_uid=20031927), [Stocks M](http://www.ncbi.nlm.nih.gov/pubmed?term=Stocks M%5BAuthor%5D&cauthor=true&cauthor_uid=20031927), [Hemmilä S](http://www.ncbi.nlm.nih.gov/pubmed?term=Hemmilä S%5BAuthor%5D&cauthor=true&cauthor_uid=20031927), [Källman T](http://www.ncbi.nlm.nih.gov/pubmed?term=Källman T%5BAuthor%5D&cauthor=true&cauthor_uid=20031927), [Zhu H](http://www.ncbi.nlm.nih.gov/pubmed?term=Zhu H%5BAuthor%5D&cauthor=true&cauthor_uid=20031927), [Zhou Y](http://www.ncbi.nlm.nih.gov/pubmed?term=Zhou Y%5BAuthor%5D&cauthor=true&cauthor_uid=20031927)F et al. (2010) Demographic histories of four spruce (Picea) species of the Qinghai-Tibetan Plateau and neighboring areas inferred from multiple nuclear loci. Mol Biol Evol 27: 1001-1014.
21. Ma XF, Szmdt AE, Wang XR (2006) Genetic structure and evolutionary history of a diploid hybrid pine *Pinus densata* inferred from the nucleotide variation at seven gene loci. Mol Biol Evol 23: 807-816.
