# Supplementary material for: Evolutionary Migration of the Disjunct Salt Cress Eutrema salsugineum (= Thellungiella salsuginea, Brassicaceae) between Asia and North America
Source: PLoS One. 2015 May 13;10(5):e0124010. doi: 10.1371/journal.pone.0124010 (PMC4430283; doi:10.1371/journal.pone.0124010)
Supplement: S12 Table — (DOC) [file pone.0124010.s014.doc]

**S12 Table.** **Population codes (N.), Species names, plant sources, and geographic origin of the sequenced material, ITS, chloroplast and nuclear loci sequenced, and GenBank accession numbers of all sequences included in this study**.

| N. | Species | Voucher | Geographic origin of the sequenced material | Nuclear sequences | | | | | | | | | | | | Chloroplast sequences | | | | | | | | | |
| --- | --- | --- | --- | --- | --- | --- | --- | --- | --- | --- | --- | --- | --- | --- | --- | --- | --- | --- | --- | --- | --- | --- | --- | --- | --- |
|  |  |  |  | *ITS*1/4 | *COP* | *DET* | *FAH* | *CHS* | *F3H* | *PGIC* | *HKT* | *RPS*1 | *RPS3* | *SOS* |  | | *psb*A*-trn*H | *trn*D*-trn*T | *ndh*F1 | *ndh*F2 | *trn*L*-trn*F | trnV | *rpo*C1 | *trn*L | *rbc*L |
| 1 | *E.salsugineum* | LiuJQ-y2007001-YJ101 | China: Kaifeng HN | KP219012 | KP208687 | KP208690 | KP208692 | KP208685 | KP208691 | KP208694 | KP208693 | KP208697 | KP208702 | KP208704 |  | | KP219015 | KP219008 | KP219006 | KP219007 | KP219009 | KP219011 | KP219005 | KP219010 | KP219004 |
| 1 | *E.salsugineum* | LiuJQ-y2007001-YJ105 | China: Kaifeng HN | KP219012 | KP208687 | KP208690 | KP208692 | KP208685 | KP208691 | KP208694 | KP208693 | KP208697 | KP208702 | KP208704 |  | | KP219015 | KP219008 | KP219006 | KP219007 | KP219009 | KP219011 | KP219005 | KP219010 | KP219004 |
| 1 | *E.salsugineum* | LiuJQ-y2007001-YJ110 | China: Kaifeng HN | KP219012 | KP208687 | KP208690 | KP208692 | KP208685 | KP208691 | KP208694 | KP208693 | KP208697 | KP208702 | KP208704 |  | | KP219015 | KP219008 | KP219006 | KP219007 | KP219009 | KP219011 | KP219005 | KP219010 | KP219004 |
| 1 | *E.salsugineum* | LiuJQ-y2007001-YJ118 | China: Kaifeng HN | KP219012 | KP208687 | KP208690 | KP208692 | KP208685 | KP208691 | KP208694 | KP208693 | KP208697 | KP208702 | KP208704 |  | | KP219015 | KP219008 | KP219006 | KP219007 | KP219009 | KP219011 | KP219005 | KP219010 | KP219004 |
| 1 | *E.salsugineum* | LiuJQ-y2007001-YJ120 | China: Kaifeng HN | KP219012 | KP208687 | KP208690 | KP208692 | KP208685 | KP208691 | KP208694 | KP208693 | KP208697 | KP208702 | KP208704 |  | | KP219015 | KP219008 | KP219006 | KP219007 | KP219009 | KP219011 | KP219005 | KP219010 | KP219004 |
| 2 | *E.salsugineum* | LiuJQ-y2007003-YJ201 | China: Lankao HN | KP219012 | KP208687 | KP208690 | KP208692 | KP208685 | KP208691 | KP208694 | KP208693 | KP208697 | KP208702 | KP208704 |  | | KP219015 | KP219008 | KP219006 | KP219007 | KP219009 | KP219011 | KP219005 | KP219010 | KP219004 |
| 2 | *E.salsugineum* | LiuJQ-y2007003-YJ202 | China: Lankao HN | KP219012 | KP208687 | KP208690 | KP208692 | KP208685 | KP208691 | KP208694 | KP208693 | KP208697 | KP208702 | KP208704 |  | | KP219015 | KP219008 | KP219006 | KP219007 | KP219009 | KP219011 | KP219005 | KP219010 | KP219004 |
| 2 | *E.salsugineum* | LiuJQ-y2007003-YJ203 | China: Lankao HN | KP219012 | KP208687 | KP208690 | KP208692 | KP208685 | KP208691 | KP208694 | KP208693 | KP208697 | KP208702 | KP208704 |  | | KP219015 | KP219008 | KP219006 | KP219007 | KP219009 | KP219011 | KP219005 | KP219010 | KP219004 |
| 2 | *E.salsugineum* | LiuJQ-y2007003-YJ205 | China: Lankao HN | KP219012 | KP208687 | KP208690 | KP208692 | KP208685 | KP208691 | KP208694 | KP208693 | KP208697 | KP208702 | KP208704 |  | | KP219015 | KP219008 | KP219006 | KP219007 | KP219009 | KP219011 | KP219005 | KP219010 | KP219004 |
| 2 | *E.salsugineum* | LiuJQ-y2007003-YJ206 | China: Lankao HN | KP219012 | KP208687 | KP208690 | KP208692 | KP208685 | KP208691 | KP208694 | KP208693 | KP208697 | KP208702 | KP208704 |  | | KP219015 | KP219008 | KP219006 | KP219007 | KP219009 | KP219011 | KP219005 | KP219010 | KP219004 |
| 3 | *E.salsugineum* | LiuJQ-y2007005-YJ307 | China: Fengqiu HN | KP219012 | KP208687 | KP208690 | KP208692 | KP208685 | KP208691 | KP208695 | KP208693 | KP208697 | KP208702 | KP208704 |  | | KP219016 | KP219008 | KP219006 | KP219007 | KP219009 | KP219011 | KP219005 | KP219010 | KP219004 |
| 3 | *E.salsugineum* | LiuJQ-y2007005-YJ308 | China: Fengqiu HN | KP219012 | KP208687 | KP208690 | KP208692 | KP208685 | KP208691 | KP208695 | KP208693 | KP208697 | KP208702 | KP208704 |  | | KP219016 | KP219008 | KP219006 | KP219007 | KP219009 | KP219011 | KP219005 | KP219010 | KP219004 |
| 3 | *E.salsugineum* | LiuJQ-y2007005-YJ310 | China: Fengqiu HN | KP219012 | KP208687 | KP208690 | KP208692 | KP208685 | KP208691 | KP208695 | KP208693 | KP208697 | KP208702 | KP208704 |  | | KP219015 | KP219008 | KP219006 | KP219007 | KP219009 | KP219011 | KP219005 | KP219010 | KP219004 |
| 4 | *E.salsugineum* | LiuJQ-y2007016-YJ405 | China: Liaocheng SD | KP219012 | KP208687 | KP208690 | KP208692 | KP208685 | KP208691 | KP208695 | KP208693 | KP208698 | KP208702 | KP208704 |  | | KP219016 | KP219008 | KP219006 | KP219007 | KP219009 | KP219011 | KP219005 | KP219010 | KP219004 |
| 4 | *E.salsugineum* | LiuJQ-y2007016-YJ407 | China: Liaocheng SD | KP219012 | KP208687 | KP208690 | KP208692 | KP208685 | KP208691 | KP208695 | KP208693 | KP208697 | KP208702 | KP208704 |  | | KP219016 | KP219008 | KP219006 | KP219007 | KP219009 | KP219011 | KP219005 | KP219010 | KP219004 |
| 4 | *E.salsugineum* | LiuJQ-y2007016-YJ408 | China: Liaocheng SD | KP219012 | KP208687 | KP208690 | KP208692 | KP208685 | KP208691 | KP208695 | KP208693 | KP208697 | KP208702 | KP208704 |  | | KP219016 | KP219008 | KP219006 | KP219007 | KP219009 | KP219011 | KP219005 | KP219010 | KP219004 |
| 4 | *E.salsugineum* | LiuJQ-y2007016-YJ409 | China: Liaocheng SD | KP219012 | KP208687 | KP208690 | KP208692 | KP208685 | KP208691 | KP208695 | KP208693 | KP208697 | KP208702 | KP208704 |  | | KP219016 | KP219008 | KP219006 | KP219007 | KP219009 | KP219011 | KP219005 | KP219010 | KP219004 |
| 4 | *E.salsugineum* | LiuJQ-y2007016-YJ410 | China: Liaocheng SD | KP219012 | KP208687 | KP208690 | KP208692 | KP208685 | KP208691 | KP208695 | KP208693 | KP208697 | KP208702 | KP208704 |  | | KP219016 | KP219008 | KP219006 | KP219007 | KP219009 | KP219011 | KP219005 | KP219010 | KP219004 |
| 5 | *E.salsugineum* | LiuJQ-y2007014-YJ502 | China: Shiping SD | KP219012 | KP208687 | KP208690 | KP208692 | KP208685 | KP208691 | KP208694 | KP208693 | KP208697 | KP208702 | KP208704 |  | | KP219016 | KP219008 | KP219006 | KP219007 | KP219009 | KP219011 | KP219005 | KP219010 | KP219004 |
| 5 | *E.salsugineum* | LiuJQ-y2007014-YJ503 | China: Shiping SD | KP219012 | KP208687 | KP208690 | KP208692 | KP208685 | KP208691 | KP208694 | KP208693 | KP208698 | KP208702 | KP208704 |  | | KP219016 | KP219008 | KP219006 | KP219007 | KP219009 | KP219011 | KP219005 | KP219010 | KP219004 |
| 5 | *E.salsugineum* | LiuJQ-y2007014-YJ505 | China: Shiping SD | KP219012 | KP208687 | KP208690 | KP208692 | KP208685 | KP208691 | KP208694 | KP208693 | KP208697 | KP208702 | KP208704 |  | | KP219016 | KP219008 | KP219006 | KP219007 | KP219009 | KP219011 | KP219005 | KP219010 | KP219004 |
| 5 | *E.salsugineum* | LiuJQ-y2007014-YJ507 | China: Shiping SD | KP219012 | KP208687 | KP208690 | KP208692 | KP208685 | KP208691 | KP208694 | KP208693 | KP208697 | KP208702 | KP208704 |  | | KP219016 | KP219008 | KP219006 | KP219007 | KP219009 | KP219011 | KP219005 | KP219010 | KP219004 |
| 5 | *E.salsugineum* | LiuJQ-y2007014-YJ508 | China: Shiping SD | KP219012 | KP208687 | KP208690 | KP208692 | KP208685 | KP208691 | KP208694 | KP208693 | KP208697 | KP208702 | KP208704 |  | | KP219016 | KP219008 | KP219006 | KP219007 | KP219009 | KP219011 | KP219005 | KP219010 | KP219004 |
| 6 | *E.salsugineum* | LiuJQ-y2007012-YJ602 | China: Qihe SD | KP219012 | KP208687 | KP208690 | KP208692 | KP208685 | KP208691 | KP208695 | KP208693 | KP208697 | KP208702 | KP208704 |  | | KP219015 | KP219008 | KP219006 | KP219007 | KP219009 | KP219011 | KP219005 | KP219010 | KP219004 |
| 6 | *E.salsugineum* | LiuJQ-y2007012-YJ604 | China: Qihe SD | KP219012 | KP208687 | KP208690 | KP208692 | KP208685 | KP208691 | KP208695 | KP208693 | KP208697 | KP208702 | KP208704 |  | | KP219015 | KP219008 | KP219006 | KP219007 | KP219009 | KP219011 | KP219005 | KP219010 | KP219004 |
| 6 | *E.salsugineum* | LiuJQ-y2007012-YJ606 | China: Qihe SD | KP219012 | KP208687 | KP208690 | KP208692 | KP208685 | KP208691 | KP208695 | KP208693 | KP208697 | KP208702 | KP208704 |  | | KP219015 | KP219008 | KP219006 | KP219007 | KP219009 | KP219011 | KP219005 | KP219010 | KP219004 |
| 6 | *E.salsugineum* | LiuJQ-y2007012-YJ608 | China: Qihe SD | KP219012 | KP208687 | KP208690 | KP208692 | KP208685 | KP208691 | KP208695 | KP208693 | KP208697 | KP208702 | KP208704 |  | | KP219015 | KP219008 | KP219006 | KP219007 | KP219009 | KP219011 | KP219005 | KP219010 | KP219004 |
| 7 | *E.salsugineum* | LiuJQ-y2007027-YJ701 | China: Boxing SD | KP219012 | KP208687 | KP208690 | KP208692 | KP208685 | KP208691 | KP208694 | KP208693 | KP208697 | KP208702 | KP208704 |  | | KP219015 | KP219008 | KP219006 | KP219007 | KP219009 | KP219011 | KP219005 | KP219010 | KP219004 |
| 7 | *E.salsugineum* | LiuJQ-y2007027-YJ704 | China: Boxing SD | KP219012 | KP208687 | KP208690 | KP208692 | KP208685 | KP208691 | KP208694 | KP208693 | KP208697 | KP208702 | KP208704 |  | | KP219015 | KP219008 | KP219006 | KP219007 | KP219009 | KP219011 | KP219005 | KP219010 | KP219004 |
| 7 | *E.salsugineum* | LiuJQ-y2007027-YJ706 | China: Boxing SD | KP219012 | KP208687 | KP208690 | KP208692 | KP208685 | KP208691 | KP208694 | KP208693 | KP208697 | KP208702 | KP208704 |  | | KP219015 | KP219008 | KP219006 | KP219007 | KP219009 | KP219011 | KP219005 | KP219010 | KP219004 |
| 8 | *E.salsugineum* | LiuJQ-y2007029-YJ805 | China: Dongying SD | KP219012 | KP208687 | KP208690 | KP208692 | KP208685 | KP208691 | KP208694 | KP208693 | KP208697 | KP208702 | KP208704 |  | | KP219016 | KP219008 | KP219006 | KP219007 | KP219009 | KP219011 | KP219005 | KP219010 | KP219004 |
| 8 | *E.salsugineum* | LiuJQ-y2007029-YJ806 | China: Dongying SD | KP219012 | KP208687 | KP208690 | KP208692 | KP208685 | KP208691 | KP208694 | KP208693 | KP208697 | KP208702 | KP208704 |  | | KP219016 | KP219008 | KP219006 | KP219007 | KP219009 | KP219011 | KP219005 | KP219010 | KP219004 |
| 8 | *E.salsugineum* | LiuJQ-y2007029-YJ807 | China: Dongying SD | KP219012 | KP208687 | KP208690 | KP208692 | KP208685 | KP208691 | KP208694 | KP208693 | KP208697 | KP208702 | KP208704 |  | | KP219015 | KP219008 | KP219006 | KP219007 | KP219009 | KP219011 | KP219005 | KP219010 | KP219004 |
| 9 | *E.salsugineum* | LiuJQ-y2007024-YJ901 | China: Binzhou SD | KP219012 | KP208687 | KP208690 | KP208692 | KP208685 | KP208691 | KP208695 | KP208693 | KP208697 | KP208702 | KP208704 |  | | KP219015 | KP219008 | KP219006 | KP219007 | KP219009 | KP219011 | KP219005 | KP219010 | KP219004 |
| 9 | *E.salsugineum* | LiuJQ-y2007024-YJ902 | China: Binzhou SD | KP219012 | KP208687 | KP208690 | KP208692 | KP208685 | KP208691 | KP208694 | KP208693 | KP208698 | KP208702 | KP208704 |  | | KP219015 | KP219008 | KP219006 | KP219007 | KP219009 | KP219011 | KP219005 | KP219010 | KP219004 |
| 9 | *E.salsugineum* | LiuJQ-y2007024-YJ904 | China: Binzhou SD | KP219012 | KP208687 | KP208690 | KP208692 | KP208685 | KP208691 | KP208695 | KP208693 | KP208697 | KP208702 | KP208704 |  | | KP219015 | KP219008 | KP219006 | KP219007 | KP219009 | KP219011 | KP219005 | KP219010 | KP219004 |
| 9 | *E.salsugineum* | LiuJQ-y2007024-YJ906 | China: Binzhou SD | KP219012 | KP208687 | KP208690 | KP208692 | KP208685 | KP208691 | KP208695 | KP208693 | KP208697 | KP208702 | KP208704 |  | | KP219015 | KP219008 | KP219006 | KP219007 | KP219009 | KP219011 | KP219005 | KP219010 | KP219004 |
| 9 | *E.salsugineum* | LiuJQ-y2007024-YJ908 | China: Binzhou SD | KP219012 | KP208687 | KP208690 | KP208692 | KP208685 | KP208691 | KP208695 | KP208693 | KP208697 | KP208702 | KP208704 |  | | KP219015 | KP219008 | KP219006 | KP219007 | KP219009 | KP219011 | KP219005 | KP219010 | KP219004 |
| 9 | *E.salsugineum* | LiuJQ-y2007024-YJ909 | China: Binzhou SD | KP219012 | KP208687 | KP208690 | KP208692 | KP208685 | KP208691 | KP208695 | KP208693 | KP208697 | KP208702 | KP208704 |  | | KP219015 | KP219008 | KP219006 | KP219007 | KP219009 | KP219011 | KP219005 | KP219010 | KP219004 |
| 10 | *E.salsugineum* | LiuJQ-y2007022-YJ1003 | China: Huimin SD | KP219012 | KP208687 | KP208690 | KP208692 | KP208685 | KP208691 | KP208695 | KP208693 | KP208697 | KP208702 | KP208704 |  | | KP219015 | KP219008 | KP219006 | KP219007 | KP219009 | KP219011 | KP219005 | KP219010 | KP219004 |
| 10 | *E.salsugineum* | LiuJQ-y2007022-YJ1004 | China: Huimin SD | KP219012 | KP208687 | KP208690 | KP208692 | KP208685 | KP208691 | KP208695 | KP208693 | KP208697 | KP208702 | KP208704 |  | | KP219015 | KP219008 | KP219006 | KP219007 | KP219009 | KP219011 | KP219005 | KP219010 | KP219004 |
| 10 | *E.salsugineum* | LiuJQ-y2007022-YJ1007 | China: Huimin SD | KP219012 | KP208687 | KP208690 | KP208692 | KP208685 | KP208691 | KP208695 | KP208693 | KP208697 | KP208702 | KP208704 |  | | KP219015 | KP219008 | KP219006 | KP219007 | KP219009 | KP219011 | KP219005 | KP219010 | KP219004 |
| 10 | *E.salsugineum* | LiuJQ-y2007022-YJ1009 | China: Huimin SD | KP219012 | KP208687 | KP208690 | KP208692 | KP208685 | KP208691 | KP208695 | KP208693 | KP208697 | KP208702 | KP208704 |  | | KP219015 | KP219008 | KP219006 | KP219007 | KP219009 | KP219011 | KP219005 | KP219010 | KP219004 |
| 10 | *E.salsugineum* | LiuJQ-y2007022-YJ1010 | China: Huimin SD | KP219012 | KP208687 | KP208690 | KP208692 | KP208685 | KP208691 | KP208695 | KP208693 | KP208697 | KP208702 | KP208704 |  | | KP219015 | KP219008 | KP219006 | KP219007 | KP219009 | KP219011 | KP219005 | KP219010 | KP219004 |
| 11 | *E.salsugineum* | LiuJQ-y2007031-YJ1101 | China: Yanshan HB | KP219012 | KP208687 | KP208690 | KP208692 | KP208685 | KP208691 | KP208695 | KP208693 | KP208698 | KP208702 | KP208704 |  | | KP219016 | KP219008 | KP219006 | KP219007 | KP219009 | KP219011 | KP219005 | KP219010 | KP219004 |
| 11 | *E.salsugineum* | LiuJQ-y2007031-YJ1103 | China: Yanshan HB | KP219012 | KP208687 | KP208690 | KP208692 | KP208685 | KP208691 | KP208695 | KP208693 | KP208698 | KP208702 | KP208704 |  | | KP219016 | KP219008 | KP219006 | KP219007 | KP219009 | KP219011 | KP219005 | KP219010 | KP219004 |
| 11 | *E.salsugineum* | LiuJQ-y2007031-YJ1105 | China: Yanshan HB | KP219012 | KP208687 | KP208690 | KP208692 | KP208685 | KP208691 | KP208695 | KP208693 | KP208698 | KP208702 | KP208704 |  | | KP219016 | KP219008 | KP219006 | KP219007 | KP219009 | KP219011 | KP219005 | KP219010 | KP219004 |
| 11 | *E.salsugineum* | LiuJQ-y2007031-YJ1109 | China: Yanshan HB | KP219012 | KP208687 | KP208690 | KP208692 | KP208685 | KP208691 | KP208695 | KP208693 | KP208698 | KP208702 | KP208704 |  | | KP219016 | KP219008 | KP219006 | KP219007 | KP219009 | KP219011 | KP219005 | KP219010 | KP219004 |
| 11 | *E.salsugineum* | LiuJQ-y2007031-YJ1110 | China: Yanshan HB | KP219012 | KP208687 | KP208690 | KP208692 | KP208685 | KP208691 | KP208695 | KP208693 | KP208698 | KP208702 | KP208704 |  | | KP219016 | KP219008 | KP219006 | KP219007 | KP219009 | KP219011 | KP219005 | KP219010 | KP219004 |
| 11 | *E.salsugineum* | LiuJQ-y2007031-YJ1114 | China: Yanshan HB | KP219012 | KP208687 | KP208690 | KP208692 | KP208685 | KP208691 | KP208695 | KP208693 | KP208698 | KP208702 | KP208704 |  | | KP219016 | KP219008 | KP219006 | KP219007 | KP219009 | KP219011 | KP219005 | KP219010 | KP219004 |
| 12 | *E.salsugineum* | LiuJQ-y2007012-YJ1201 | China: Botou HB | KP219012 | KP208687 | KP208690 | KP208692 | KP208685 | KP208691 | KP208694 | KP208693 | KP208698 | KP208702 | KP208704 |  | | KP219016 | KP219008 | KP219006 | KP219007 | KP219009 | KP219011 | KP219005 | KP219010 | KP219004 |
| 12 | *E.salsugineum* | LiuJQ-y2007012-YJ1202 | China: Botou HB | KP219012 | KP208687 | KP208690 | KP208692 | KP208685 | KP208691 | KP208694 | KP208693 | KP208698 | KP208702 | KP208704 |  | | KP219016 | KP219008 | KP219006 | KP219007 | KP219009 | KP219011 | KP219005 | KP219010 | KP219004 |
| 12 | *E.salsugineum* | LiuJQ-y2007012-YJ1203 | China: Botou HB | KP219012 | KP208687 | KP208690 | KP208692 | KP208685 | KP208691 | KP208694 | KP208693 | KP208698 | KP208702 | KP208704 |  | | KP219016 | KP219008 | KP219006 | KP219007 | KP219009 | KP219011 | KP219005 | KP219010 | KP219004 |
| 12 | *E.salsugineum* | LiuJQ-y2007012-YJ1206 | China: Botou HB | KP219012 | KP208687 | KP208690 | KP208692 | KP208685 | KP208691 | KP208694 | KP208693 | KP208698 | KP208702 | KP208704 |  | | KP219016 | KP219008 | KP219006 | KP219007 | KP219009 | KP219011 | KP219005 | KP219010 | KP219004 |
| 12 | *E.salsugineum* | LiuJQ-y2007012-YJ1207 | China: Botou HB | KP219012 | KP208687 | KP208690 | KP208692 | KP208685 | KP208691 | KP208694 | KP208693 | KP208698 | KP208702 | KP208704 |  | | KP219016 | KP219008 | KP219006 | KP219007 | KP219009 | KP219011 | KP219005 | KP219010 | KP219004 |
| 13 | *E.salsugineum* | LiuJQ-y2007037-YJ1302 | China: Raoyang HB | KP219012 | KP208687 | KP208690 | KP208692 | KP208685 | KP208691 | KP208694 | KP208693 | KP208698 | KP208702 | KP208704 |  | | KP219015 | KP219008 | KP219006 | KP219007 | KP219009 | KP219011 | KP219005 | KP219010 | KP219004 |
| 13 | *E.salsugineum* | LiuJQ-y2007037-YJ1303 | China: Raoyang HB | KP219012 | KP208687 | KP208690 | KP208692 | KP208685 | KP208691 | KP208694 | KP208693 | KP208698 | KP208702 | KP208704 |  | | KP219015 | KP219008 | KP219006 | KP219007 | KP219009 | KP219011 | KP219005 | KP219010 | KP219004 |
| 13 | *E.salsugineum* | LiuJQ-y2007037-YJ1304 | China: Raoyang HB | KP219012 | KP208687 | KP208690 | KP208692 | KP208685 | KP208691 | KP208694 | KP208693 | KP208698 | KP208702 | KP208704 |  | | KP219015 | KP219008 | KP219006 | KP219007 | KP219009 | KP219011 | KP219005 | KP219010 | KP219004 |
| 13 | *E.salsugineum* | LiuJQ-y2007037-YJ1305 | China: Raoyang HB | KP219012 | KP208687 | KP208690 | KP208692 | KP208685 | KP208691 | KP208694 | KP208693 | KP208698 | KP208702 | KP208704 |  | | KP219015 | KP219008 | KP219006 | KP219007 | KP219009 | KP219011 | KP219005 | KP219010 | KP219004 |
| 14 | *E.salsugineum* | LiuJQ-y2007048-YJ1401 | China: Leting HB | KP219012 | KP208687 | KP208690 | KP208692 | KP208685 | KP208691 | KP208694 | KP208693 | KP208698 | KP208702 | KP208704 |  | | KP219015 | KP219008 | KP219006 | KP219007 | KP219009 | KP219011 | KP219005 | KP219010 | KP219004 |
| 14 | *E.salsugineum* | LiuJQ-y2007048-YJ1402 | China: Leting HB | KP219012 | KP208687 | KP208690 | KP208692 | KP208685 | KP208691 | KP208695 | KP208693 | KP208698 | KP208702 | KP208704 |  | | KP219015 | KP219008 | KP219006 | KP219007 | KP219009 | KP219011 | KP219005 | KP219010 | KP219004 |
| 14 | *E.salsugineum* | LiuJQ-y2007048-YJ1403 | China: Leting HB | KP219012 | KP208687 | KP208690 | KP208692 | KP208685 | KP208691 | KP208694 | KP208693 | KP208698 | KP208702 | KP208704 |  | | KP219016 | KP219008 | KP219006 | KP219007 | KP219009 | KP219011 | KP219005 | KP219010 | KP219004 |
| 15 | *E.salsugineum* | LiuJQ-y2007041-YJ1501 | China: Qingguang TJ | KP219012 | KP208687 | KP208690 | KP208692 | KP208685 | KP208691 | KP208694 | KP208693 | KP208697 | KP208702 | KP208704 |  | | KP219016 | KP219008 | KP219006 | KP219007 | KP219009 | KP219011 | KP219005 | KP219010 | KP219004 |
| 15 | *E.salsugineum* | LiuJQ-y2007041-YJ1502 | China: Qingguang TJ | KP219012 | KP208687 | KP208690 | KP208692 | KP208685 | KP208691 | KP208694 | KP208693 | KP208698 | KP208702 | KP208704 |  | | KP219016 | KP219008 | KP219006 | KP219007 | KP219009 | KP219011 | KP219005 | KP219010 | KP219004 |
| 15 | *E.salsugineum* | LiuJQ-y2007041-YJ1503 | China: Qingguang TJ | KP219012 | KP208687 | KP208690 | KP208692 | KP208685 | KP208691 | KP208694 | KP208693 | KP208697 | KP208702 | KP208704 |  | | KP219015 | KP219008 | KP219006 | KP219007 | KP219009 | KP219011 | KP219005 | KP219010 | KP219004 |
| 16 | *E.salsugineum* | LiuJQ-y2007047-YJ1601 | China: Wuqing TJ | KP219012 | KP208687 | KP208690 | KP208692 | KP208685 | KP208691 | KP208695 | KP208693 | KP208698 | KP208702 | KP208704 |  | | KP219015 | KP219008 | KP219006 | KP219007 | KP219009 | KP219011 | KP219005 | KP219010 | KP219004 |
| 16 | *E.salsugineum* | LiuJQ-y2007047-YJ1602 | China: Wuqing TJ | KP219012 | KP208687 | KP208690 | KP208692 | KP208685 | KP208691 | KP208695 | KP208693 | KP208698 | KP208702 | KP208704 |  | | KP219015 | KP219008 | KP219006 | KP219007 | KP219009 | KP219011 | KP219005 | KP219010 | KP219004 |
| 16 | *E.salsugineum* | LiuJQ-y2007047-YJ1603 | China: Wuqing TJ | KP219012 | KP208687 | KP208690 | KP208692 | KP208685 | KP208691 | KP208695 | KP208693 | KP208698 | KP208702 | KP208704 |  | | KP219015 | KP219008 | KP219006 | KP219007 | KP219009 | KP219011 | KP219005 | KP219010 | KP219004 |
| 16 | *E.salsugineum* | LiuJQ-y2007047-YJ1609 | China: Wuqing TJ | KP219012 | KP208687 | KP208690 | KP208692 | KP208685 | KP208691 | KP208695 | KP208693 | KP208698 | KP208702 | KP208704 |  | | KP219015 | KP219008 | KP219006 | KP219007 | KP219009 | KP219011 | KP219005 | KP219010 | KP219004 |
| 16 | *E.salsugineum* | LiuJQ-y2007047-YJ1610 | China: Wuqing TJ | KP219012 | KP208687 | KP208690 | KP208692 | KP208685 | KP208691 | KP208695 | KP208693 | KP208698 | KP208702 | KP208704 |  | | KP219015 | KP219008 | KP219006 | KP219007 | KP219009 | KP219011 | KP219005 | KP219010 | KP219004 |
| 17 | *E.salsugineum* | LiuJQ-y2013051-YJ1702 | China: Manasi XJ | KP219012 | KP208688 | KP208690 | KP208692 | KP208686 | KP208691 | KP208695 | KP208693 | KP208697 | KP208703 | KP208704 |  | | KP219017 | KP219008 | KP219006 | KP219007 | KP219009 | KP219011 | KP219005 | KP219010 | KP219004 |
| 17 | *E.salsugineum* | LiuJQ-y2013051-YJ1703 | China: Manasi XJ | KP219012 | KP208688 | KP208690 | KP208692 | KP208686 | KP208691 | KP208695 | KP208693 | KP208697 | KP208703 | KP208704 |  | | KP219017 | KP219008 | KP219006 | KP219007 | KP219009 | KP219011 | KP219005 | KP219010 | KP219004 |
| 17 | *E.salsugineum* | LiuJQ-y2013051-YJ1704 | China: Manasi XJ | KP219012 | KP208688 | KP208690 | KP208692 | KP208686 | KP208691 | KP208695 | KP208693 | KP208697 | KP208703 | KP208704 |  | | KP219017 | KP219008 | KP219006 | KP219007 | KP219009 | KP219011 | KP219005 | KP219010 | KP219004 |
| 17 | *E.salsugineum* | LiuJQ-y2013051-YJ1705 | China: Manasi XJ | KP219012 | KP208688 | KP208690 | KP208692 | KP208686 | KP208691 | KP208695 | KP208693 | KP208697 | KP208703 | KP208704 |  | | - | - | - | - | - | - | - | - | - |
| 17 | *E.salsugineum* | LiuJQ-y2013051-YJ1708 | China: Manasi XJ | KP219012 | KP208688 | KP208690 | KP208692 | KP208686 | KP208691 | KP208695 | KP208693 | KP208697 | KP208703 | KP208704 |  | | - | - | - | - | - | - | - | - | - |
| 17 | *E.salsugineum* | LiuJQ-y2013051-YJ1710 | China: Manasi XJ | KP219012 | KP208688 | KP208690 | KP208692 | KP208686 | KP208691 | KP208695 | KP208693 | KP208697 | KP208703 | KP208704 |  | | - | - | - | - | - | - | - | - | - |
| 18 | *E.salsugineum* | LiuJQ-y2013052-YJ1801 | Russian: Altai | KP219012 | KP208688 | KP208690 | KP208692 | KP208686 | KP208691 | KP208695 | KP208693 | KP208697 | KP208703 | KP208704 |  | | KP219018 | KP219008 | KP219006 | KP219007 | KP219009 | KP219011 | KP219005 | KP219010 | KP219004 |
| 18 | *E.salsugineum* | LiuJQ-y2013052-YJ1802 | Russian: Altai | KP219012 | KP208688 | KP208690 | KP208692 | KP208686 | KP208691 | KP208695 | KP208693 | KP208697 | KP208703 | KP208704 |  | | KP219018 | KP219008 | KP219006 | KP219007 | KP219009 | KP219011 | KP219005 | KP219010 | KP219004 |
| 18 | *E.salsugineum* | LiuJQ-y2013052-YJ1803 | Russian: Altai | KP219012 | KP208688 | KP208690 | KP208692 | KP208686 | KP208691 | KP208695 | KP208693 | KP208697 | KP208703 | KP208704 |  | | KP219018 | KP219008 | KP219006 | KP219007 | KP219009 | KP219011 | KP219005 | KP219010 | KP219004 |
| 19 | *E.salsugineum* | LiuJQ-y2013053-YJ1902 | Russian: Tuva | KP219012 | KP208689 | KP208690 | KP208692 | KP208686 | KP208691 | KP208695 | KP208693 | KP208699 | KP208702 | KP208704 |  | | KP219019 | KP219008 | KP219006 | KP219007 | KP219009 | KP219011 | KP219005 | KP219010 | KP219004 |
| 19 | *E.salsugineum* | LiuJQ-y2013053-YJ1903 | Russian: Tuva | KP219012 | KP208689 | KP208690 | KP208692 | KP208686 | KP208691 | KP208695 | KP208693 | KP208699 | KP208702 | KP208704 |  | | KP219019 | KP219008 | KP219006 | KP219007 | KP219009 | KP219011 | KP219005 | KP219010 | KP219004 |
| 19 | *E.salsugineum* | LiuJQ-y2013053-YJ1905 | Russian: Tuva | KP219012 | KP208689 | KP208690 | KP208692 | KP208686 | KP208691 | KP208695 | KP208693 | KP208699 | KP208702 | KP208704 |  | | KP219019 | KP219008 | KP219006 | KP219007 | KP219009 | KP219011 | KP219005 | KP219010 | KP219004 |
| 19 | *E.salsugineum* | LiuJQ-y2013053-YJ1907 | Russian: Tuva | KP219012 | KP208689 | KP208690 | KP208692 | KP208686 | KP208691 | KP208695 | KP208693 | KP208699 | KP208702 | KP208704 |  | | KP219019 | KP219008 | KP219006 | KP219007 | KP219009 | KP219011 | KP219005 | KP219010 | KP219004 |
| 19 | *E.salsugineum* | LiuJQ-y2013053-YJ1908 | Russian: Tuva | KP219012 | - | - | - | - | - | - | - | - | - | - |  | | KP219019 | KP219008 | KP219006 | KP219007 | KP219009 | KP219011 | KP219005 | KP219010 | KP219004 |
| 20 | *E.salsugineum* | LiuJQ-y2013054-YJ2001 | Russian: Buriatia | KP219012 | KP208687 | KP208690 | KP208692 | KP208686 | KP208691 | KP208696 | KP208693 | KP208701 | KP208702 | KP208704 |  | | KP453987 | KP219008 | KP219006 | KP219007 | KP219009 | KP219011 | KP219005 | KP219010 | KP219004 |
| 20 | *E.salsugineum* | LiuJQ-y2013054-YJ2004 | Russian: Buriatia | KP219012 | KP208687 | KP208690 | KP208692 | KP208686 | KP208691 | KP208696 | KP208693 | KP208701 | KP208702 | KP208704 |  | | KP453987 | KP219008 | KP219006 | KP219007 | KP219009 | KP219011 | KP219005 | KP219010 | KP219004 |
| 20 | *E.salsugineum* | LiuJQ-y2013054-YJ2005 | Russian: Buriatia | KP219012 | KP208687 | KP208690 | KP208692 | KP208686 | KP208691 | KP208696 | KP208693 | KP208701 | KP208702 | KP208704 |  | | KP453987 | KP219008 | KP219006 | KP219007 | KP219009 | KP219011 | KP219005 | KP219010 | KP219004 |
| 20 | *E.salsugineum* | LiuJQ-y2013054-YJ2006 | Russian: Buriatia | KP219012 | KP208687 | KP208690 | KP208692 | KP208686 | KP208691 | KP208696 | KP208693 | KP208701 | KP208702 | KP208704 |  | | - | - | - | - | - | - | - | - | - |
| 21 | *E.salsugineum* | LiuJQ-y2013055-YJ2101 | Russian: Yakutsk | KP219012 | KP208689 | KP208690 | KP208692 | KP208686 | KP208691 | KP208695 | KP208693 | KP208697 | KP208703 | KP208704 |  | | KP219016 | KP219008 | KP219006 | KP219007 | KP219009 | KP219011 | KP219005 | KP219010 | KP219004 |
| 21 | *E.salsugineum* | LiuJQ-y2013055-YJ2104 | Russian: Yakutsk | KP219012 | KP208689 | KP208690 | KP208692 | KP208686 | KP208691 | KP208695 | KP208693 | KP208697 | KP208703 | KP208704 |  | | KP219016 | KP219008 | KP219006 | KP219007 | KP219009 | KP219011 | KP219005 | KP219010 | KP219004 |
| 21 | *E.salsugineum* | LiuJQ-y2013055-YJ2105 | Russian: Yakutsk | KP219012 | KP208689 | KP208690 | KP208692 | KP208686 | KP208691 | KP208695 | KP208693 | KP208697 | KP208703 | KP208704 |  | | KP219016 | KP219008 | KP219006 | KP219007 | KP219009 | KP219011 | KP219005 | KP219010 | KP219004 |
| 22 | *E.salsugineum* | LiuJQ-y2013056-YJ2202 | Canada: Yukon | KP219012 | KP208689 | KP208690 | KP208692 | KP208686 | KP208691 | KP208695 | KP208693 | KP208700 | KP208703 | KP208704 |  | | KP219016 | KP219008 | KP219006 | KP219007 | KP219009 | KP219011 | KP219005 | KP219010 | KP219004 |
| 22 | *E.salsugineum* | LiuJQ-y2013056-YJ2205 | Canada: Yukon | KP219012 | KP208689 | KP208690 | KP208692 | KP208686 | KP208691 | KP208695 | KP208693 | KP208700 | KP208703 | KP208704 |  | | KP219016 | KP219008 | KP219006 | KP219007 | KP219009 | KP219011 | KP219005 | KP219010 | KP219004 |
| 22 | *E.salsugineum* | LiuJQ-y2013056-YJ2206 | Canada: Yukon | KP219012 | KP208689 | KP208690 | KP208692 | KP208686 | KP208691 | KP208695 | KP208693 | KP208700 | KP208703 | KP208704 |  | | KP219016 | KP219008 | KP219006 | KP219007 | KP219009 | KP219011 | KP219005 | KP219010 | KP219004 |
| 22 | *E.salsugineum* | LiuJQ-y2013056-YJ2208 | Canada: Yukon | KP219012 | - | - | - | - | - | - | - | - | - | - |  | | KP219016 | KP219008 | KP219006 | KP219007 | KP219009 | KP219011 | KP219005 | KP219010 | KP219004 |
| 22 | *E.salsugineum* | LiuJQ-y2013056-YJ2210 | Canada: Yukon | KP219012 | - | - | - | - | - | - | - | - | - | - |  | | KP219016 | KP219008 | KP219006 | KP219007 | KP219009 | KP219011 | KP219005 | KP219010 | KP219004 |
| 23 | *E.salsugineum* | LiuJQ-y2013057-YJ2301 | Canada: Cracker Creek | KP219012 | KP208689 | KP208690 | KP208692 | KP208686 | KP208691 | KP208695 | KP208693 | KP208700 | KP208703 | KP208704 |  | | KP219016 | KP219008 | KP219006 | KP219007 | KP219009 | KP219011 | KP219005 | KP219010 | KP219004 |
| 23 | *E.salsugineum* | LiuJQ-y2013057-YJ2302 | Canada: Cracker Creek | KP219012 | KP208689 | KP208690 | KP208692 | KP208686 | KP208691 | KP208695 | KP208693 | KP208700 | KP208703 | KP208704 |  | | KP219016 | KP219008 | KP219006 | KP219007 | KP219009 | KP219011 | KP219005 | KP219010 | KP219004 |
| 23 | *E.salsugineum* | LiuJQ-y2013057-YJ2303 | Canada: Cracker Creek | KP219012 | KP208689 | KP208690 | KP208692 | KP208686 | KP208691 | KP208695 | KP208693 | KP208700 | KP208703 | KP208704 |  | | KP219016 | KP219008 | KP219006 | KP219007 | KP219009 | KP219011 | KP219005 | KP219010 | KP219004 |
| 24 | *E.salsugineum* | LiuJQ-y2013058-YJ2402 | Canada: Dilibrough | KP219012 | KP208689 | KP208690 | KP208692 | KP208686 | KP208691 | KP208695 | KP208693 | KP208700 | KP208703 | KP208704 |  | | KP219016 | KP219008 | KP219006 | KP219007 | KP219009 | KP219011 | KP219005 | KP219010 | KP219004 |
| 24 | *E.salsugineum* | LiuJQ-y2013058-YJ2403 | Canada: Dilibrough | KP219012 | KP208689 | KP208690 | KP208692 | KP208686 | KP208691 | KP208695 | KP208693 | KP208700 | KP208703 | KP208704 |  | | KP219016 | KP219008 | KP219006 | KP219007 | KP219009 | KP219011 | KP219005 | KP219010 | KP219004 |
| 24 | *E.salsugineum* | LiuJQ-y2013058-YJ2407 | Canada: Dilibrough | KP219012 | KP208689 | KP208690 | KP208692 | KP208686 | KP208691 | KP208695 | KP208693 | KP208700 | KP208703 | KP208704 |  | | KP219016 | KP219008 | KP219006 | KP219007 | KP219009 | KP219011 | KP219005 | KP219010 | KP219004 |
| 24 | *E.salsugineum* | LiuJQ-y2013058-YJ2409 | Canada: Dilibrough | KP219012 | - | - | - | - | - | - | - | - | - | - |  | | KP219016 | KP219008 | KP219006 | KP219007 | KP219009 | KP219011 | KP219005 | KP219010 | KP219004 |
| 25 | *E.halophila* | LiuJQ-y2013065-YJ2503 | Kazakhstan: Pavlodar | KP219013 | - | - | - | - | - | - | - | - | - | - |  | | KP453986 | - | - | - | - | - | - | - | - |
| 25 | *E.halophila* | LiuJQ-y2013065-YJ2505 | Kazakhstan: Pavlodar | KP219013 | - | - | - | - | - | - | - | - | - | - |  | | KP453986 | - | - | - | - | - | - | - | - |
| 25 | *E.halophila* | LiuJQ-y2013065-YJ2507 | Kazakhstan: Pavlodar | KP219013 | - | - | - | - | - | - | - | - | - | - |  | | KP453986 | - | - | - | - | - | - | - | - |
| 25 | *E.halophila* | LiuJQ-y2013065-YJ2509 | Kazakhstan: Pavlodar | KP219013 | - | - | - | - | - | - | - | - | - | - |  | | KP453986 | - | - | - | - | - | - | - | - |
| 25 | *E.halophila* | LiuJQ-y2013065-YJ2510 | Kazakhstan: Pavlodar | KP219013 | - | - | - | - | - | - | - | - | - | - |  | | KP453986 | - | - | - | - | - | - | - | - |
| 26 | *E.botschantzevii* | LiuJQ-y2013066-YJ2601 | Russian: Saratov | KP219014 | - | - | - | - | - | - | - | - | - | - |  | | KP453985 | - | - | - | - | - | - | - | - |
| 26 | *E.botschantzevii* | LiuJQ-y2013066-YJ2603 | Russian: Saratov | KP219014 | - | - | - | - | - | - | - | - | - | - |  | | KP453985 | - | - | - | - | - | - | - | - |
| 26 | *E.botschantzevii* | LiuJQ-y2013066-YJ2604 | Russian: Saratov | KP219014 | - | - | - | - | - | - | - | - | - | - |  | | KP453985 | - | - | - | - | - | - | - | - |
| 26 | *E.botschantzevii* | LiuJQ-y2013066-YJ2608 | Russian: Saratov | KP219014 | - | - | - | - | - | - | - | - | - | - |  | | KP453985 | - | - | - | - | - | - | - | - |
| 26 | *E.botschantzevii* | LiuJQ-y2013066-YJ2609 | Russian: Saratov | KP219014 | - | - | - | - | - | - | - | - | - | - |  | | KP453985 | - | - | - | - | - | - | - | - |
